# Urinary metabolomic investigations in vitiligo patients

**DOI:** 10.1038/s41598-020-75135-0

**Published:** 2020-10-22

**Authors:** Wei Liu, Xiao-Yan Liu, Yue-Tong Qian, Dong-Dong Zhou, Jia-Wei Liu, Tian Chen, Wei Sun, Dong-Lai Ma

**Affiliations:** 1 Department of Dermatology, Peking Union Medical College Hospital, Chinese Academy of Medical Sciences, National Clinical Research Center for Skin and Immune Diseases, Beijing, 100730 China; 2 Institute of Basic Medical Sciences, Chinese Academy of Medical Sciences, School of Basic Medicine, Peking Union Medical College, Beijing, 100005 China

**Keywords:** Immunology, Biomarkers, Diseases

## Abstract

Urinary metabolomics is a useful non-invasive tool for large-scale screening of disease-related metabolites. However, no comprehensive urinary metabolomic analysis of vitiligo is presently available. To investigate the urine metabolic pattern of vitiligo patients, we conducted a combined cross-sectional and prospective self-control cohort study and an untargeted urinary metabolomic analysis. In the cross-sectional study, 295 vitiligo patients and 192 age‐ and sex‐matched controls were enrolled, and 71 differential metabolites between two groups were identified. Pathway enrichment analysis revealed that drug metabolism-cytochrome P450, biopterin metabolism, vitamin B9 (folate) metabolism, selenoamino acid metabolism, and methionine and cysteine metabolism showed significant enrichment in vitiligo patients compared with the status in healthy controls. In the self-control cohort, 46 active vitiligo patients were recruited to analyse the urinary metabolic signatures after treatment. All of these patients were asked to undertake follow-up visits every 2 months three times after first consulting and the disease stage was evaluated compared with that at the last visit. Folate metabolism, linoleate metabolism, leukotriene metabolism, alkaloid biosynthesis, and tyrosine metabolism were predicted to be involved in vitiligo activity. Our study is the first attempt to reveal urinary metabolic signatures of vitiligo patients and provides new insights into the metabolic mechanisms of vitiligo.

## Introduction

Vitiligo is a common acquired pigmentary disorder characterised by depigmentation of skin resulting from the destruction of epidermal melanocytes. The incidence of vitiligo has been estimated to be 1% of the global population^1^. It has major impacts on patients’ social activity and mental health, causing severe distress. Unlike other cutaneous disorders, no erythema or scaling is present in vitiligo lesions. In some atypical or early-stage cases of vitiligo, it is quite difficult to diagnose and differentiate from other hypopigmented diseases^2,3^. This may cause a delay in treatment at an early stage and some patients with other diseases can even be misdiagnosed with vitiligo^4^. Moreover, the course of vitiligo is unpredictable and it is difficult to assess the treatment response at an early stage^5–7^, which means that treatment evaluation is often postponed. Therefore, a biomarker helping physicians to objectively recognise the atypical lesions, follow patients over time, or accurately determine the treatment response at an early stage would be of great value^6,8,9^.

Many groups have attempted to find vitiligo biomarkers. Clinical signs such as Koebner phenomenon, blurred border, and confetti-like depigmentation have been described as clinical markers of active vitiligo, but these signs only present in a subset of vitiligo patients and are not sufficiently objective^10,11^. Skin tissue biomarkers have been reported, such as basal cell vacuolisation^12^, CD8^+^ lymphocyte infiltration^13^, and increased expression of heat shock protein-70^14^, CXCL9^15^, and sCD25^16^. However, skin biopsy is a traumatic examination and it is difficult to apply to patients with more than one active period. Moreover, an overlap in histological findings has been found between active and stable vitiligo^9^.

Blood biomarkers of vitiligo, including soluble CDs (sCD25, sCD27)^6^, chemokines (CXCL9, CXCL10)^17^, S100B^11^, cytokines (IL-1β, IL-10, IL-17)^18,19^, and homocysteine^20^, have been reported to be related to the occurrence and activity of vitiligo. However, these markers still need further clinical verification and different studies on them have even shown contrasting results^9^. Thus, there is a need for more study to find non-invasive biomarkers that could help to diagnose and monitor the stage of vitiligo.

Metabolomics is a widely used biological approach for identifying and measuring the changes in biological samples^21,22^. Urine examinations are considered as non-invasive, rapid diagnostic methods that have been used in scientific research and clinical applications. Recent studies have shown that urine metabolomics has become a useful method to identify biomarkers for some skin diseases, such as psoriasis^23^, dermatomyositis^24^, melanoma^25^, syphilis^26^, and atopic dermatitis^27^. However, to date, few studies focusing on changes in urine metabolites in vitiligo patients have been performed. Previous metabolic studies mostly focused on a few metabolites in vitiligo, such as urinary catecholamines and vanillylmandelic acid on a small scale^28,29^. However, no comprehensive urinary metabolomic analysis of vitiligo is currently available.

**Figure 1:**
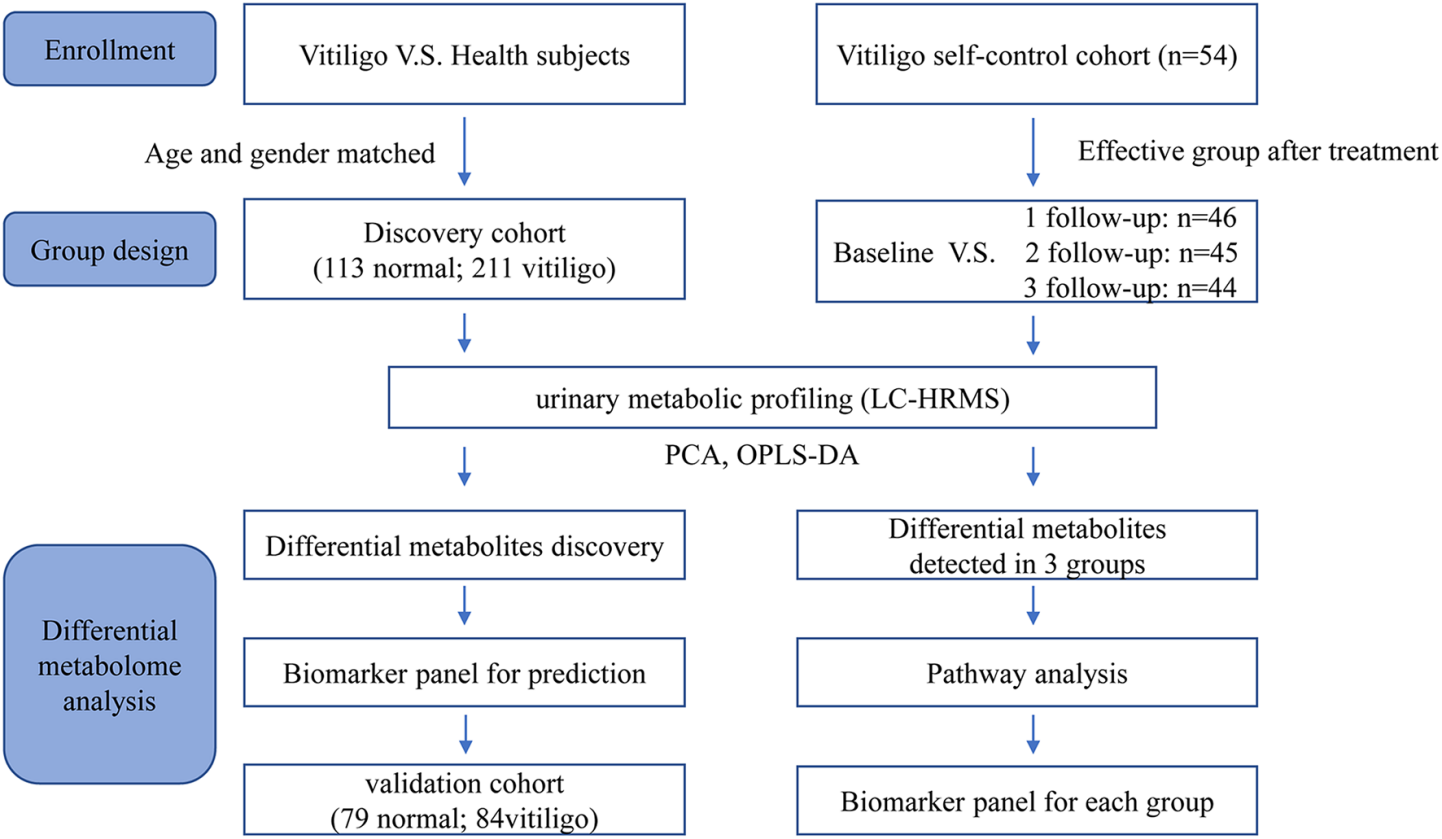
The work flow of this study.

In this study we conducted a urine metabolomic analysis in a cross-sectional and prospective self-control cohort, and attempted to identify the metabolic biomarkers for both the diagnosis and the treatment response of vitiligo patients. We first analysed the urine samples from 295 Chinese vitiligo sufferers who volunteered to participate and 192 healthy controls, along with investigating the metabolic features and biomarkers of the vitiligo patient group. Then, in the self-control cohort, we targeted the active patients and investigated the variation in urine metabolites after treatment. The analysis contributes to our ability to diagnose this disease, revealing metabolic changes involved in disease activity, and helping to identify the urinary metabolic patterns in different effective stages and potential biomarkers for treatment responses (Fig. 1). Our study is the first attempt to reveal urinary metabolic signatures of vitiligo patients. These results might be helpful to explore the metabolic changes involved in the pathogenesis of vitiligo, and to diagnose this disease and monitor its treatment response in a clinical setting.

## Results

### Clinical characteristics

#### Discovery cohort and validation cohort

Urine samples (midstream) were randomly divided into a discovery cohort of 211 vitiligo patients and 113 age- and sex-matched healthy human adults, and a validation cohort of 84 vitiligo patients and 79 healthy human adults. Among the 295 vitiligo patients, 19 segmental vitiligo patients were enrolled. Their detailed demographics and disease subtypes are shown in Tables 1 and S1.

**Table 1:** Demographics of healthy subjects and vitiligo patients enrolled in this study.

	DHC (n = 113)	DVC (n = 211)	p*	VHC (n = 79)	VVC (n = 84)	*p*^*#*^	SAV (n = 46)
Average age (years)	25.18 ± 15.61	23.03 ± 13.53	0.26	24.38 ± 14.16	23.21 ± 13.28	0.97	32.7 ± 12.45
**Sex**			0.48			0.64	
Female	55	94		48	48		19
Male	58	117		31	36		27

DHC: Discovery cohort healthy control. DVC: Discovery cohort vitiligo cases. VHC: Validation cohort healthy control. VVC: Validation cohort vitiligo cases. SAV: Self-control active vitiligo cohort.

*p-value of Chi-square test comparing DHC with DVC.

^#^*p*-value of Chi-square test comparing VHC with VVC.

#### Self-control cohort

Forty-six active vitiligo patients were recruited at Peking Union Medical College Hospital. Among these patients, 44 patients undertook the first follow-up visit (2 months after the first consultation), 44 patients undertook the second follow-up visit (4 months after the first consultation), and 43 patients undertook the third follow-up visit (6 months after the first consultation; for more details, see Tables 1 and S1). By comparing the digital follow-up photographs, wood lamp images, and clinical examination results obtained at the last visit, 104 effective/improved visit points were recorded. Then, we performed metabolic profiling between the different follow-up visits and the baseline among the patients in whom treatment was effective to further investigate whether metabolic profiles could reflect improvement of vitiligo and detect the urine metabolites with a tendency to change in association with this.

### Urine metabolomic pattern of vitiligo patients compared with healthy controls

#### Metabolites differentially expressed between vitiligo patients and healthy controls were identified

**Figure 2:**
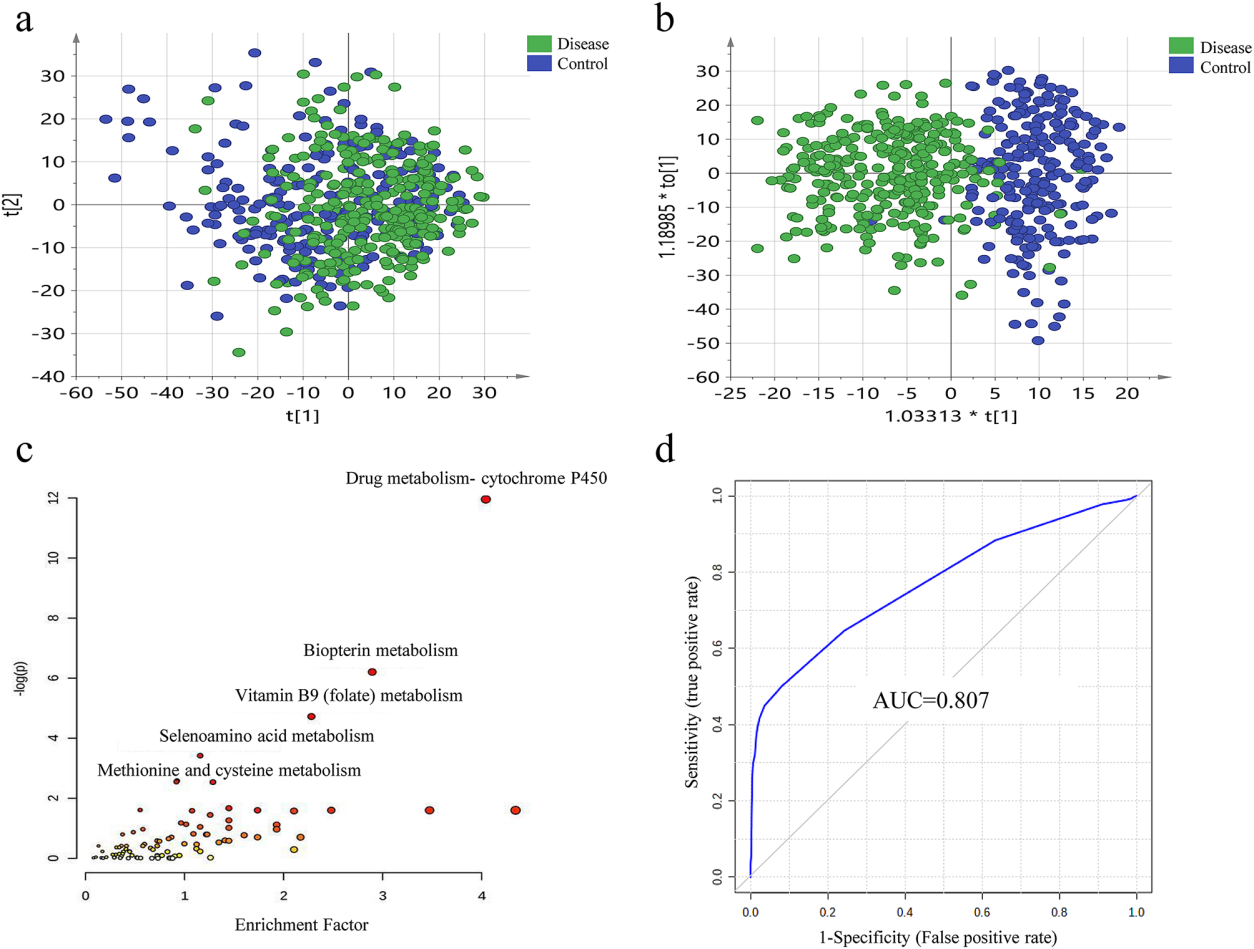
Analysis of urine metabolome between vitiligo patients and healthy controls. (**a**) PCA analysis of urine metabolome. (SIMCA 14.0 software, Umetrics, Sweden) (**b**) Score plot of OPLS-DA model between vitiligo patients and healthy controls showed a better separation. (SIMCA 14.0 software, Umetrics, Sweden) (**c**) Pathway enrichment analysis showed significant enrichment (p < 0.05) of several pathways between the two groups. (MetaboAnalyst 3.0, https://www.metaboanalyst.ca) (**d**) A metabolites panel consisting of 7alpha-hydroxy-3-oxochol-4-en-24-oic acid, deoxyuridine, 3,4-octadienoylglycine, threoninyl-Proline showed the predictive ability with an AUC of 0.807 in the validation cohort. (MetaboAnalyst 3.0, https://www.metaboanalyst.ca).

To eliminate the potential confounders between the vitiligo and healthy groups, subjects were gender- and age-matched. LC–MS-based urine samples from vitiligo patients and healthy controls yielded 2500 features after quality control (QC) filtering. Tight clustering of the QC samples indicated good repeatability of analysis (Fig. S1). The principal component analysis (PCA) score plot did not show an obvious trend of separation between the vitiligo patients and the healthy controls (Fig. 2a). However, the orthogonal partial least squares analysis (OPLS-DA) model achieved better separation (Fig. 2b). Permutation tests were carried out to confirm the stability and robustness of the supervised models presented in this study (Fig. S2a). Differential metabolites were assigned based on VIP values > 1 and adjusted *p*-values < 0.05. In total, 71 differentially expressed metabolites were identified, with 61 metabolites upregulated and 10 downregulated in the vitiligo group compared with the levels in the healthy controls (Table S2). Further metabolic comparison between the 19 segmental vitiligo samples and 19 age- and gender-matched nonsegmental vitiligo samples showed that the two disease types had no significant differences of urine metabolomics (Fig. S2c,d).

Pathway enrichment analysis showed significant enrichment (*p*-values < 0.05) of several pathways in the vitiligo group compared with the case in healthy controls, including drug metabolism-cytochrome P450, biopterin metabolism, vitamin B9 (folate) metabolism, selenoamino acid metabolism, and methionine and cysteine metabolism (Fig. 2c, Table 2).

**Table 2:** Differential metabolic pathways that may involve in vitiligo pathogenesis.

Pathways	Possible involved function	References
**Vitiligo patients V.S. healthy controls**		
biopterin metabolism	Oxidative stress Melanin biosynthesis	^30–32^
Vitamin B9 (folate) metabolism	Melanocyte destruction Oxidative stress Interleukin 6 production Tyrosinase inhibition Triggering autoimmunity and nuclear factor κB (NF-κB) activation	^33–36^
Selenoamino acid metabolism	Oxidative stress	^37,38^
Methionine and cysteine metabolism	Oxidative stress	^37,38^
**Active vitiligo self-control cohort**		
Linoleate metabolism	Immune modulation and response Alter membrane fluidity Change activities of antigen Receptors	^39–41^
Leukotriene metabolism	Melanocyte migration	^42^
Alkaloid biosynthesis	Promote melanocyte proliferation stimulate repigmentation	^7,43^
Tyrosine metabolism	Melanin synthesis	

#### A metabolite panel consisting of 7a-hydroxy-3-oxochol-4-en-24-oic acid, deoxyuridine, 3,4-octadienoylglycine, and threoninyl-proline showed the best predictive ability

The diagnostic accuracy of the identified metabolites with differential expression in vitiligo compared with that in the healthy controls was further evaluated. A total of 26 metabolites had potential diagnostic value with an AUC above 0.7, and four metabolites had good diagnostic value with an AUC above 0.8. A multivariate ROC curve-based exploratory analysis was performed to achieve a better predictive model using a logistic regression algorithm. As a result, a metabolite panel consisting of 7a-hydroxy-3-oxochol-4-en-24-oic acid, deoxyuridine, 3,4-octadienoylglycine, and threoninyl-proline showed the best predictive ability with an AUC of 0.818 for the testing data (Fig. S2b). In the validation cohort, this panel had an AUC of 0.807 (Fig. 2d).

### Metabolomic characterisation of vitiligo treatment monitoring

#### In the effective group after treatment, the metabolic change became more stable over time

To discover potential biomarkers for monitoring vitiligo after treatment, urine metabolomics at baseline visits and that at the visits with improved vitiligo during the three follow-ups were compared. Unsupervised PCA was first used to show the tendency for separation of urine metabolites between the baseline visits and the three follow-up visits with improved vitiligo. Furthermore, OPLS-DA was used to select metabolites associated with vitiligo improvement. For the first follow-up, the PCA score plot showed a significant overlap (Fig. S3a) and OPLS-DA showed no statistically significant difference between the two groups (Fig. S3b).

**Figure 3:**
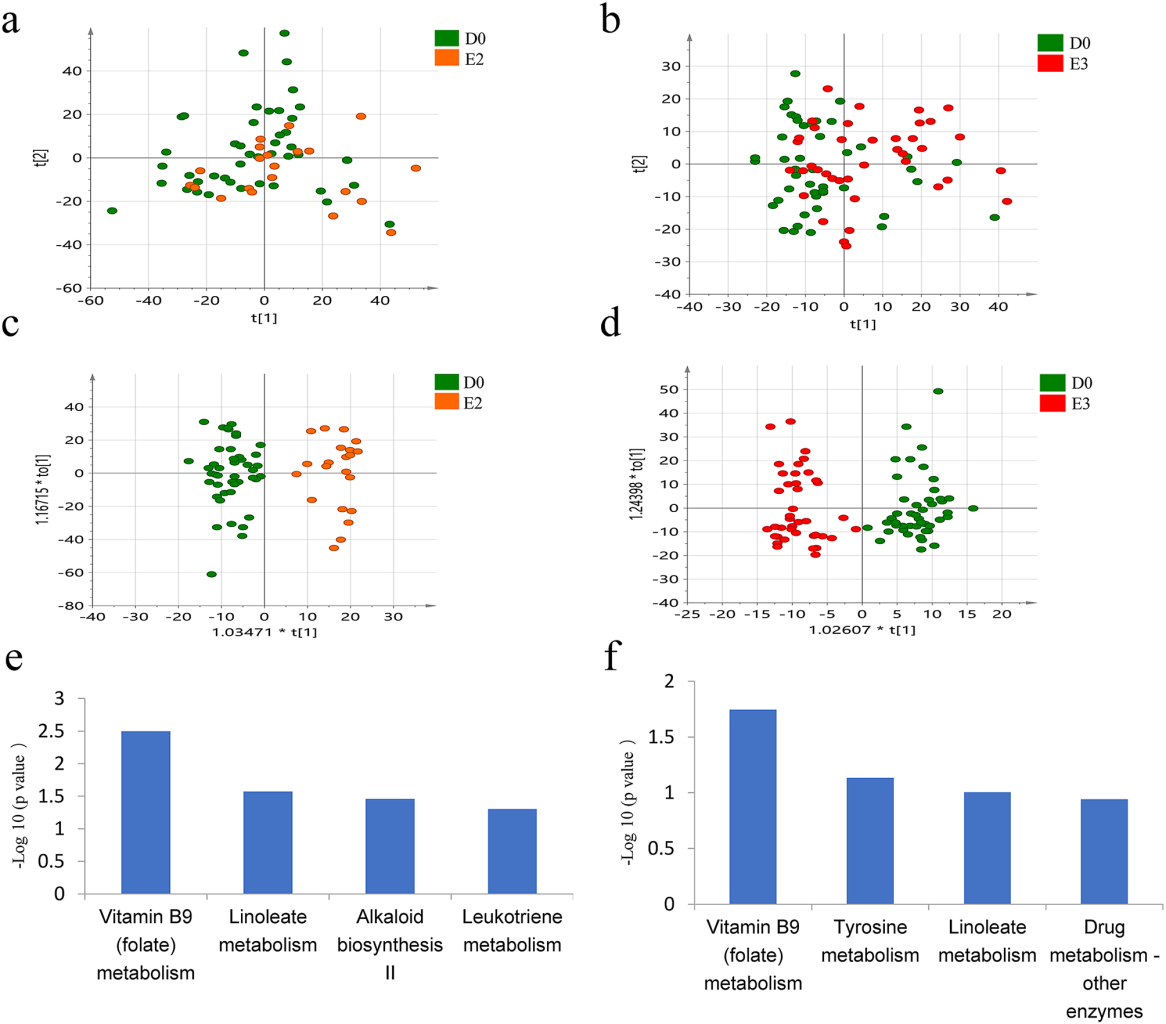
Analysis of urine metabolome in vitiligo longitudinal self-control cohort. (**a**,**b**) PCA analysis of urine metabolome compared between baseline and second follow-up samples (**a**), baseline and second follow-up samples (**b**). (SIMCA 14.0 software, Umetrics, Sweden) (**c**,**d**) Score plot of OPLS-DA model based on metabolome between baseline and second follow-up samples (**c**), baseline and second follow-up samples (**d**). (SIMCA 14.0 software, Umetrics, Sweden) (**e**,**f**) Pathway enrichment analysis between baseline and second follow-up samples (**e**), baseline and second follow-up samples (**f**). (MetaboAnalyst 3.0, https://www.metaboanalyst.ca).

However, for the second follow-up (after treatment for 4 months), the PCA score plot showed slight separation between baseline and the second follow-up visit (Fig. 3a). An OPLS-DA plot showed separation with R2Y = 0.71, Q2 = 0.33, and *p*-value of 1.40535e−006 (Fig. 3c). A total of 41 differential metabolites were detected, all of which had an AUC > 0.7 (Table S3). Pathway analysis showed enrichment of pathways, including folate metabolism, linoleate metabolism, leukotriene metabolism, and alkaloid biosynthesis II in second follow-up group (Fig. 3e, Table 2).

For the third follow-up (after treatment for 6 months), the PCA score plot showed slight separation between baseline and the third follow-up visit (Fig. 3b). An OPLS-DA plot showed separation with R2Y = 0.71, Q2 = 0.33, and *p*-value of 5.67251e−011 (Fig. 3d). A total of 42 differentially expressed metabolites were identified, all of which had an AUC > 0.7 (Table S3). Pathway analysis showed enrichment of pathways including folate metabolism, linoleate metabolism, drug metabolism-other enzymes, and tyrosine metabolism in third follow-up group (Fig. 3f, Table 2).

#### Metabolite intensity presented different signatures in different follow-up samples

**Figure 4:**
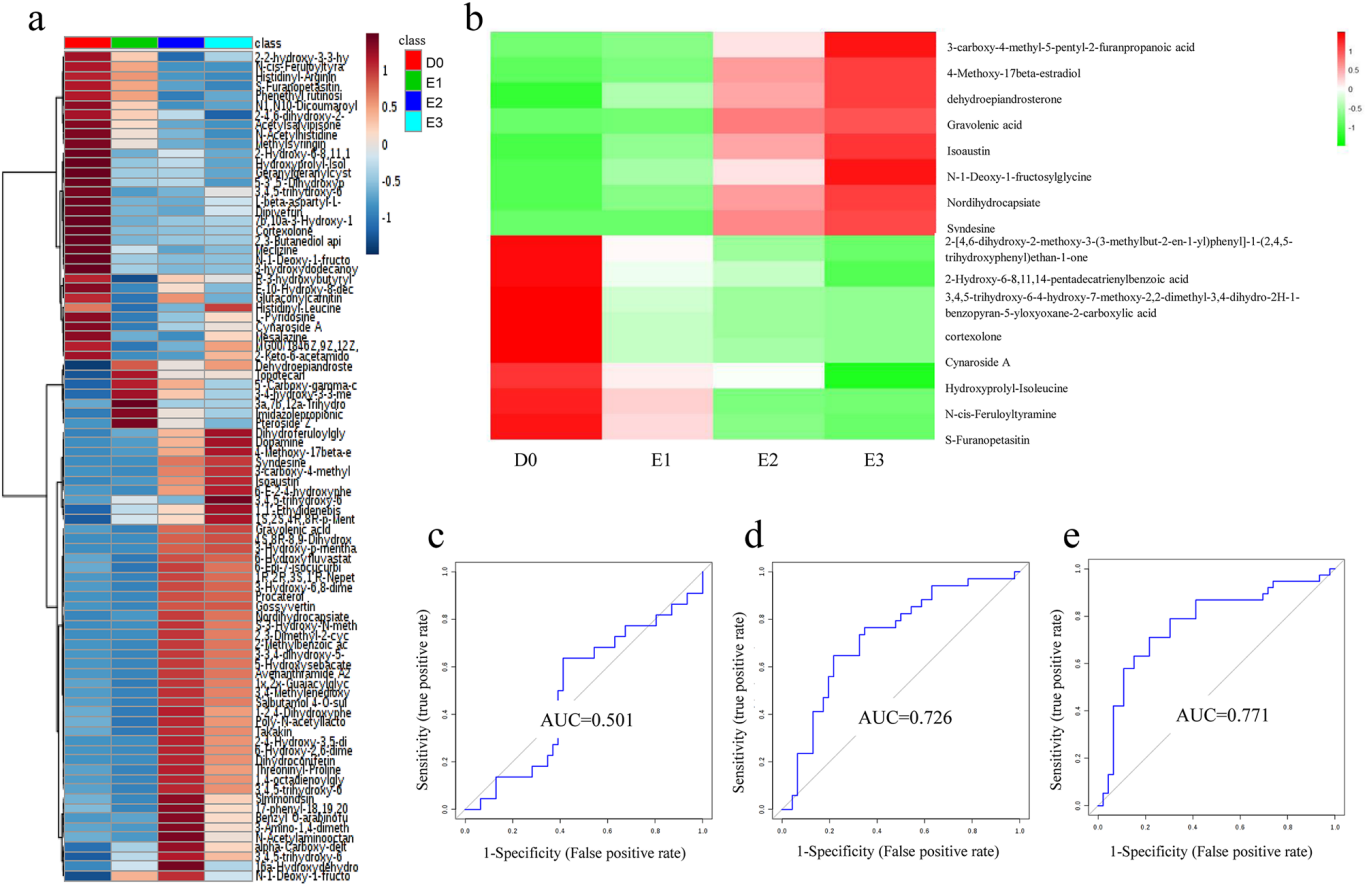
Analysis of metabolic profiling in effective vitiligo self-control group. (**a**) Relative intensity of differential metabolites at different visits. (MetaboAnalyst 3.0, https://www.metaboanalyst.ca) (**b**) Sixteen of the metabolites showed a gradually increasing or decreasing trend with the improvement of the disease, with 8 gradually elevating and 8 gradually decreasing. (R package of pheatmap, R.3.4.2) (**c**–**e**) A panel consisting of 4-Methoxy-17beta-estradiol, dehydroepiandrosterone and cortexolone was used for predictive model construction using logistic regression. The ROCs were analyzed between baseline and first follow-up samples (**c**), baseline and second follow-up samples (**d**), baseline and third follow-up samples (**e**). (MetaboAnalyst 3.0, https://www.metaboanalyst.ca).

Overall, 86 differential metabolites were identified between the baseline visits and the three follow-up visits with active vitiligo. The relative intensity of these metabolites was plotted as a heatmap to show the pathways enriched in each group (Fig. 4a). In the baseline group, metabolites involved in lysine degradation and cortexolone metabolism were upregulated. In the first follow-up visit, metabolites involved in bile acid biosynthesis and androsterone metabolism were upregulated. Notably, the metabolic signature in the second and third follow-up groups changed dramatically compared with that in the baseline samples. In the second follow-up visit, metabolites involved in tyrosine metabolism and androsterone biosynthesis were upregulated, while in the third follow-up visit, those involved in steroid hormone biosynthesis and glycine metabolism were upregulated.

#### Sixteen metabolites showed a gradually increasing or decreasing trend as the disease improved

Metabolites showing a gradually increasing or decreasing trend with the improvement of the disease would have potential value for the active monitoring of vitiligo treatment. Here, 16 metabolites showed gradual variations, with eight gradually elevating and eight gradually declining (Fig. 4b). A panel consisting of 4-methoxy-17b-estradiol, dehydroepiandrosterone, and cortexolone was used for predictive model construction using logistic regression. The discrimination ability of this biomarker panel was tested in different follow-up groups. The results showed that this panel had no predictive ability in the samples from the first follow-up with an AUC of 0.501. However, in the samples from the second and third follow-ups, this panel had a gradually increasing predictive value of 0.726 and 0.771, respectively (Fig. 4c–e). These results may indicate that the urinary metabolic pattern varies in different stages after effective treatment. In the early stage, metabolomics cannot reflect the disease response, but shows gradually increasing predictive value over time. Permutation tests were carried out to confirm the stability and robustness of the supervised models in different follow-up samples (Fig. S3c–e).

## Discussion

### Metabolite signatures in vitiligo diagnosis

We performed urine metabolomic analysis to screen out vitiligo-related metabolites. In total, 71 metabolites, including lipids, amino acids, peptides, organic acid, and bile acids, were found to be differentially expressed in vitiligo patients compared with that in healthy controls. Pathway enrichment analysis showed significant enrichment of several pathways in vitiligo patients, including drug metabolism-cytochrome P450, biopterin metabolism, vitamin B9 (folate) metabolism, selenoamino acid metabolism, and methionine and cysteine metabolism. The pathways reported to be involved in vitiligo are compared with our results in Table 2.

In vitiligo patients, biopterin synthesis/recycling/regulation process defects were reported to be involved in the pathogenesis of this condition from at least two aspects^30–32^. On the one hand, H_2_O_2_ can directly oxidise (6R)-l-erythro-5,6,7,8-tetrahydrobiopterin to 6-biopterin, which is cytotoxic to melanocytes in vitro^31^. On the other hand, low 4a-hydroxy-tetrahydrobiopterin dehydratase activity and the accumulation of 7-tetrahydrobiopterin were also detected in vitiligo patients, leading to the competitive inhibition of epidermal phenylalanine hydroxylase and influencing melanin synthesis^32^. Our results showed that the biopterin metabolism pathway showed significant enrichment in vitiligo patients compared with the status in healthy controls, which is consistent with the published literature and indicates disordered biopterin metabolism in the pathogenesis of vitiligo.

Homocysteine metabolism is dependent on the vitamin B_12_ and folate pathway, and the levels of these three compounds were reported to be interconnected^33^. Previous studies reported that folate metabolism and homocysteine metabolism are involved in the occurrence and activity of vitiligo^33–35^. Homocysteine may mediate melanocyte destruction via increased oxidative damage, interleukin-6 production, and tyrosinase inhibition, triggering autoimmunity and nuclear factor κB activation, and then contributing to the occurrence of vitiligo^33,36^. Our study also suggested that folate metabolism is involved in vitiligo, but the specific mechanism needs further clarification.

### Vitiligo treatment monitoring using urine metabolomics

The post-treatment longitudinal metabolomic analysis in vitiligo patients for whom treatment was effective showed that the urinary metabolic pattern varied in the different effective stages. In the early effective stage (within 2 months), metabolomics could not reflect the disease response, but it showed gradually increasing predictive value over time, from 4 to 6 months after treatment. Differential metabolites were detected by comparing different follow-up samples and baseline samples. The enrichment of pathways including linoleate metabolism, leukotriene metabolism, alkaloid biosynthesis II, and tyrosine metabolism was found. These have also been previously reported to be related to vitiligo (Table 2).

The alkaloid biosynthesis pathway was found to be related to disease activity in our study. A previous study showed that piperine, the main alkaloid from *Piper nigrum* fruit, can induce morphological alterations in melanin cells, with more and longer dendrites observed^43^. Another recent study showed the piperine can promote melanocyte proliferation and stimulate repigmentation in vitiligo patients^7^. We also found that (S)-3-hydroxy-*N*-methylcoclaurine, an intermediate in isoquinoline alkaloid biosynthesis, was present at higher levels in effective stages than in the initial active stage in our study. These results indicate that alkaloid may contribute to the improvement of vitiligo, but the specific mechanism involved needs further investigation.

Our results showed that, among the 16 metabolites having a gradually increasing or decreasing trend with disease improvement, the panel of dehydroepiandrosterone (DHEA), cortexolone, and 4-methoxy-17b-estradiol showed the best predictive value. Interestingly, these three metabolites are all related to steroid hormones in the body. Gurpinar et al*.* reported that vitiligo patients significantly differed from the healthy population in terms of hormones and psychological distress^44^. In addition, lymphocyte DNA from vitiligo patients is sensitive to steroid-generated electrophiles^45^. These findings indicate that steroid hormones are probably involved in the pathogenesis of vitiligo.

DHEA is known as a stress-related hormone and has antioxidant properties^44,46^. Gurpinar et al*.* reported that an abnormal hormonal response to stress lowers DHEAS (sulphated form of DHEA) in vitiligo patients, which is a possible mechanism behind the development of this condition^44^. They also assumed that low levels of DHEAS may be a reason for a longer duration of illness or a worse response to treatment in vitiligo patients^44^. This is consistent with our finding that DHEA levels gradually increased as the disease improved.

Cortexolone is the precursor of cortisol. It has been shown that there was a significant increase in the serum level of cortisol in patients with active vitiligo compared with that in those with either stable vitiligo or a healthy control group^47^. This may be related to high levels of depression and psychogenic stress in vitiligo patients, which can stimulate the secretion of corticotropin-releasing hormone, thus inducing or exacerbating depigmented lesions^48–50^. Our findings are consistent with previous reports showing that cortexolone levels gradually decreased as the disease improved. The above results indicate that a high level of cortisol is probably related to the active disease stage.

4-Methoxy-17b-estradiol is a member of the oestrogens and their derivatives. Ranson et al. reported that oestradiol can increase the tyrosinase activity of human melanocytes, which may be beneficial to vitiligo patients^51^. However, oestrogen also inhibits melanocyte growth and induces the immune response against melanocytes, leading to depigmentation^47,52^. Further study has shown that oestrogens contribute to oxidative stress via H_2_O_2_ and lead to DNA damage in vitiligo patients^45^. In our study, the levels of 4-methoxy-17b-estradiol gradually increased as the disease improved, which revealed that 4-methoxy-17b-estradiol might be an effective metabolic biomarker in vitiligo patients. These results illustrate that neurological, psychological, and endocrine disorders also play an important role in vitiligo formation and progression.

In conclusion, in this study, we investigated urine metabolomic features in vitiligo patients. We screened out 71 vitiligo-related metabolites by comparison with healthy controls and found that pathways including drug metabolism-cytochrome P450, biopterin metabolism, vitamin B9 (folate) metabolism, selenoamino acid metabolism, methionine, and cysteine metabolism showed significant enrichment in vitiligo patients. In the post-treatment longitudinal metabolomic analysis of vitiligo patients for whom treatment was effective, differential urinary metabolites were detected and the relative intensity of these metabolites was plotted. Folate metabolism, linoleate metabolism, leukotriene metabolism, alkaloid biosynthesis, and tyrosine metabolism were predicted to participate in vitiligo activity. This is the first attempt at applying urine metabolomics to vitiligo, which provides new insights into vitiligo diagnosis, phase evaluation, treatment monitoring, and related mechanisms.

## Materials and methods

### Ethics

This study was approved by the ethics committee of Peking Union Medical College Hospital, Chinese Academy of Medical Sciences, and Peking Union Medical College (No. JS-2146). Informed consent was obtained from all the participants (both patients and healthy subjects) and also from the legal guardians of participants less than 18 years of age. The methods were carried out in accordance with the guidance of the National Key Research and Development Program of China and the National Natural Science Foundation of China.

### Discovery cohort and validation cohort

Urine samples (midstream) were collected on an empty stomach from 295 vitiligo patients and 192 healthy adults at Peking Union Medical College Hospital between March 1, 2016, and February 20, 2019 (Table S1). The diagnosis of vitiligo was confirmed by two experienced dermatologists according to the typical clinical presentation of depigmented lesions and wood lamp images. Patients and controls were excluded if they had suffered from any acute conditions in the last 3 months or been diagnosed with urinary system tumours. Once collected, the urine samples were stored at − 80 °C as soon as possible.

### Self-control cohort

In accordance with published reports, a longitudinal self-control cohort was designed^53^. Forty-six active nonsegmental vitiligo patients (with the emergence of new lesions or the enlargement of original lesions within 6 months according to the Vitiligo Disease Activity score^54^) were enrolled at Peking Union Medical College Hospital between May 1, 2016, and December 20, 2018. The diagnosis of vitiligo and assessment of disease activity were accomplished by two experienced dermatologists according to the typical clinical presentation of depigmented lesions and wood lamp images. All patients were initially given prednisone tablets at 0.5 mg/kg/day orally and the dose was decreased gradually within 4–5 weeks. After the first urine sample had been collected at the first visit as a baseline, we asked the patients to undertake a follow-up visit every 2 months three times. Urine samples were collected at every visit and were stored at − 80 °C as soon as possible. By comparing the digital follow-up photographs, wood lamp images, and clinical examination results obtained at the last visit, the patients showing an effective response after treatment were recorded (with no new lesion appearance and original lesions showed repigmentation). Patients were excluded if they had suffered from acute conditions in the last 3 months, had been diagnosed with urinary system tumours, or had any contraindications for systemic prednisone.

### Sample preparation

Urine sample preparation was performed based on a previously described method^55^. In brief, acetonitrile (200 μL) was added to each urine sample (200 μL); then, the mixture was vortexed for 30 s and centrifuged at 14,000 g for 10 min. The supernatant was dried under a vacuum and then reconstituted with 200 μL of 2% acetonitrile. Urinary metabolites were further separated from larger molecules using 10 kDa molecular weight cut-off ultracentrifugation filters (Millipore Amicon Ultra, MA) before transfer to the autosamplers. The QC sample was a pooled urine sample prepared by mixing aliquots of 50 representative samples across different groups to be analysed and was therefore globally representative of the whole sample set. One in every ten of the QC samples was injected throughout the analytical run to provide a set of data from which method stability and repeatability could be assessed.

### LC–MS analysis

HRLC-MS was selected for urinary metabolite detection due to its high sensitivity and reproducibility. Urine metabolite separation and analysis were conducted using a Waters ACQUITY H-class LC system coupled with an LTQ-Orbitrap Velos Pro mass spectrometer (Thermo Fisher Scientific, MA). The following 18-min gradient on a Waters HSS C18 column (3.0 × 100 mm, 1.7 μm) at a flow rate of 0.5 mL/min was used: 0–1 min, 2% solvent B (mobile phase A: 0.1% formic acid in H_2_O; mobile phase B: acetonitrile); 1–3 min, 2%–55% solvent B; 3–8 min, 55%–100% solvent B; 8–13 min, 100% solvent B; 13–13.1 min, 100%–2% solvent B; 13.1–18 min, 2% solvent B. The column temperature was set at 45 °C.

All samples were fully scanned from 100 to 1000 m/z at a resolution of 60 K. The automatic gain control (AGC) target was 1 × 10^6^, and the maximum injection time (IT) was 100 ms. The extracted MS features were divided into several targeted lists and imported to the MS2 method for targeted data-dependent analysis. MS/MS fragment acquisition was performed at a resolution of 15 K with an AGC target of 5 × 10^5^. Collision energy was optimised as 20, 40, or 60 for each targeted list with higher-energy collisional dissociation (HCD) fragmentation. The injection order of urine samples was randomised to reduce any experimental bias. The QC sample was injected regularly to monitor system stability.

### Statistical analysis

Raw data files were processed by Progenesis QI (Waters, Milford, MA) software based on a previously published identification strategy, which included sample alignment, peak picking, peak grouping, deconvolution, and final information export^56^ (Supplementary Method). The exported data were further preprocessed by MetaboAnalyst 3.0 (https://www.metaboanalyst.ca), which included missing value estimation, log transformation, and Pareto scaling. Variables that were missed in 50% or more of the samples were removed from further statistical analysis.

Nonparametric tests (Wilcoxon rank-sum tests) were used to evaluate the significance of variables related to disease using MetaboAnalyst 3.0 (https://www.metaboanalyst.ca). Benjamini–Hochberg correction was applied throughout to account for multiple test comparisons. An FDR cut-off of 0.05 was applied. Pattern recognition analysis (PCA and OPLS-DA) was carried out using SIMCA 14.0 software (Umetrics, Sweden) to visualise group classification and select significant features. A total of 100 permutation tests were used to validate the OPLS-DA model to avoid over-fitting of the model. Significantly different metabolites were chosen according to the following criteria: (i) adjusted p-value < 0.05 and (ii) variable importance plot (VIP) value obtained from OPLS-DA greater than 1^57^. Metabolic pathway enrichment analysis was performed using the Mummichog algorithm by MetaboAnalyst 3.0 (https://www.metaboanalyst.ca). Heatmaps were visualised using the MetaboAnalyst 3.0 (https://www.metaboanalyst.ca) and R package of pheatmap (R.3.4.2). Those variables showing significant differences (VIP > 1, adjusted p-value < 0.05) between E1 (first follow-up visit of the effective group), E2 (second follow-up visit of the effective group), or E3 (third follow-up visit of the effective group) and D0 (baseline visit before treatment) were defined as variables reflecting an effective response during different treatment stages. In addition, those variables showing trends of a single change (increase or decrease) along the treatment course (E1 to E3) were defined as potential markers for monitoring the treatment effect and potential variables showing biological significance in vitiligo. A multivariate ROC curve-based exploratory analysis was performed using a logistic regression algorithm by MetaboAnalyst 3.0 (https://www.metaboanalyst.ca).

### Feature annotation and metabolite identification

MS1 features were divided into several targeted lists and imported to the MS method for targeted data-dependent analysis. The MS/MS spectra were further imported to Progenesis QI for database searching (HMDB: https://www.hmdb.ca/) and MS/MS spectral matching using the “MetFrag” algorithm^58^.

Detailed compound identification information (.csv file) included compound ID, adducts, formula, score, MS/MS score, mass error (in ppm), isotope similarity, theoretical isotope distribution, web link, and m/z values. Confirmation of the different compounds was performed using the parameters, including score, fragmentation score, and isotope similarity given by Progenesis QI. A score ranging from 0 to 60 was used to quantify the reliability of each identity. According to the score results of the reference standards, the threshold was set at 35.0. Fragmentation score represents the degree of matching between the theoretical fragments and the measured ones. A fragmentation score of 0 indicates that no match occurs or the compound generates no fragments. Isotope similarity is calculated by comparison of the measured isotope distribution of a precursor ion with the theoretical one. The compound identification is more reliable when higher values are obtained.

## Supplementary information

Supplementary Methods.

Supplementary Figures.

Supplementary Table 1.

Supplementary Table 2.

Supplementary Table 3.
