# Supplementary material for: Urinary metabolomic investigations in vitiligo patients
Source: Sci Rep. 2020 Oct 22;10:17989. doi: 10.1038/s41598-020-75135-0 (PMC7582886; doi:10.1038/s41598-020-75135-0)
Supplement: Supplementary file 1 — Supplementary Methods. [file 41598_2020_75135_MOESM1_ESM.docx]

**Title: Urinary metabolomic investigations in vitiligo patients**

Author: Wei Liu^1^, Xiao-Yan Liu^2^, Yue-Tong Qian^1^, Dong-Dong Zhou^2^, Jia-Wei Liu^1^, Tian Chen^1^, Wei Sun^2*^, Dong-Lai Ma^1*^

1. Department of Dermatology, Peking Union Medical College Hospital, Chinese Academy of Medical Sciences, National Clinical Research Center for Skin and Immune Diseases, Beijing 100730, China.
2. Institute of Basic Medical Sciences, Chinese Academy of Medical Sciences, School of Basic Medicine, Peking Union Medical College, Beijing, 100005, China.

***** Correspondence: [mdonglai@sohu.com, 86-10-69151543 (DLM)](mailto:mdonglai@sohu.com,%2086-10-69151543%20(DLM));

[sunwei1018@sina.com](mailto:sunwei1018@sina.com), 86-10-69156995 (SW)

**1. Data processing using Progenesis QI**

The detailed workflow for data processing facilitated by Progenesis QI is involved “create a new experiment”, “import data”, “review alignment”, “experiment design setup”, “peak picking”, “reviewed convolution”, and “identify compounds” in sequence. In general, the whole process ran automatically using optimized parameter settings. (1) In the stage of create a new experiment, adduct ion was carefully selected as it would influence the number of characterized compounds and also the identification accuracy. Based on the ionization behaviors of reference standards, the adduct ion forms, comprising [M + H]+, [M + Na]+, [M + K], [M + NH4]+, [2M + H]+, [2M + Na]+,[2M + NH4]+, [M + H – H2O]+ and [M + H – 2H2O]+ , were selected. (2) The MS data acquired by LC-MS for all the URINE samples were imported into the Progenesis QI software, generating a 2D ion intensity map with the retention time and m/z information as the ordinate and abscissa, respectively. (3) Peak alignment was carried out in automatic manner taking a QC run as the reference, the score values for all the samples were greater than 90 %. (4) For peak picking, the thresholds of chromatographic peak absolute intensity, and retention time limits can be set to achieve the maximum real ion signals with noise excluded. In the present study, absolute intensity and retention time limit were set at 1000 and default. (5) Further compound identification was performed by searching the HMDB database (2018 version). The MS1 mass tolerance was set as 10 ppm and the MS/MS mass tolerance was set as 20 ppm.

**2. Permutations test**

Permutations test was used to assess the risk that the OPLS-DA model is spurious. The idea of this validation is to compare the goodness of fit (R2 and Q2) of the original model with the goodness of fit of several models based on data where the order of the Y-observations has been randomly permuted, while the X-matrix has been kept intact. The plot shows, for a selected Y-variable, on the vertical axis the values of R2 and Q2 for the original model (far to the right) and of the Y-permuted models further to the left. The horizontal axis shows the correlation between the permuted Y-vectors and the original Y-vector for the selected Y. The original Y has the correlation 1.0 with itself, defining the high point on the horizontal axis. The plot above strongly indicates that the original model is valid. The criteria for validity are: (1) All blue Q2-values to the left are lower than the original points to the right. or (2) The blue regression line of the Q2-points intersects the vertical axis (on the left) at, or below zero. (referred from “Simca14 software”). According to these criteria, models in present study are valid^5^. Herein, 100 permutations were performed using simca 14.0 software.
