# Supplementary material for: Urinary metabolomic investigations in vitiligo patients
Source: Sci Rep. 2020 Oct 22;10:17989. doi: 10.1038/s41598-020-75135-0 (PMC7582886; doi:10.1038/s41598-020-75135-0)
Supplement: Supplementary file 2 — Supplementary Figures. [file 41598_2020_75135_MOESM2_ESM.pdf]

a

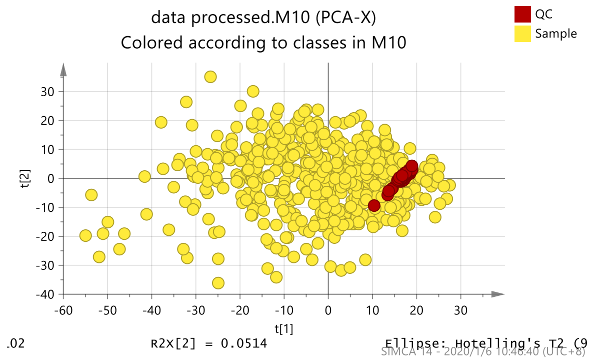

Supplementary figure 1. (a) PCA analysis of urine metabolome between vitiligo patients and healthy controls.

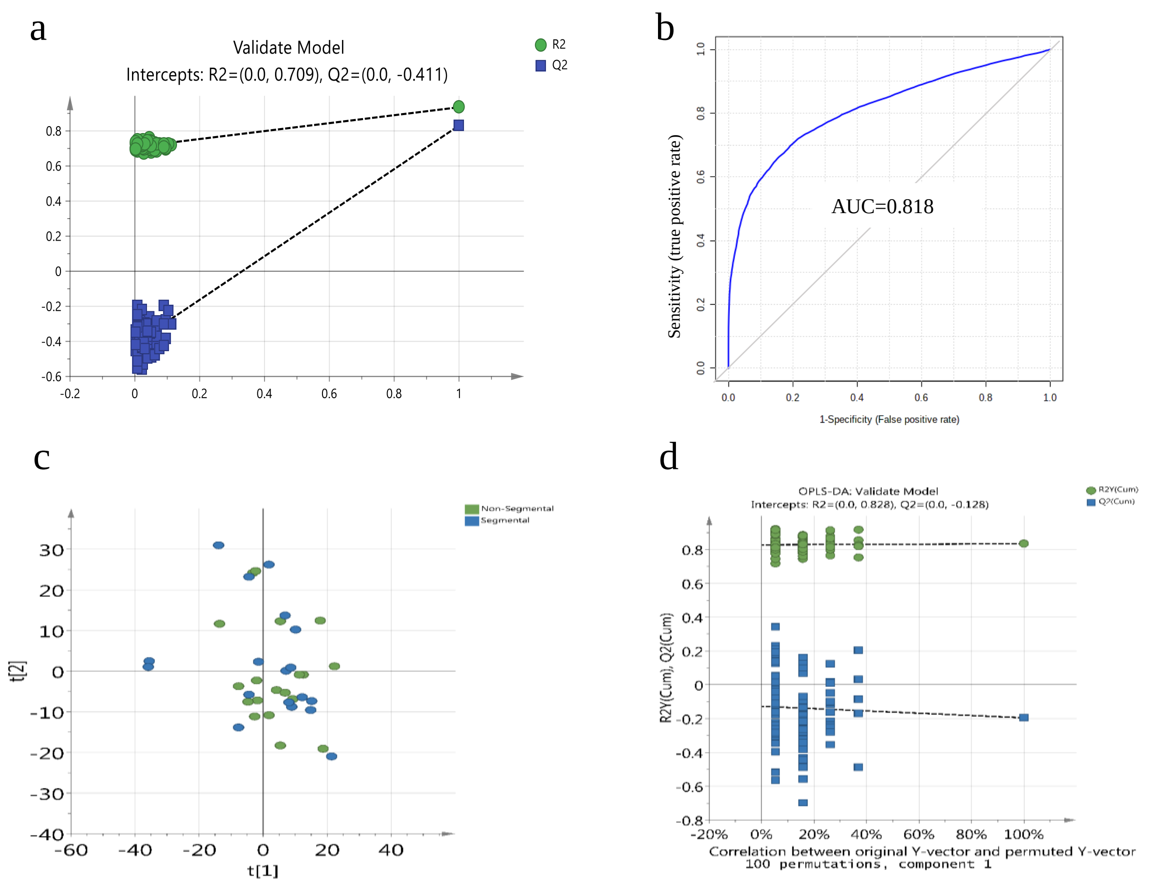

Supplementary figure 2. Analysis of urine metabolome between vitiligo patients and healthy controls. (a) 100 permutation validation plot for the OPLS-DA model. (b) A metabolites panel consisting of 7α–hydroxy-3-oxochol-4-en-24-oic acid, deoxyuridine, 3,4-octadienoylglycine, threoninyl-Proline showed the best predictive ability with an AUC of 0.82 for the testing dataset and 0.808 for the 10-fold cross –validation. (c) PCA analysis of urine metabolome between segmental vitiligo patients and non-segmental vitiligo patients. (d) 100 permutation validation plot for the OPLS-DA model.

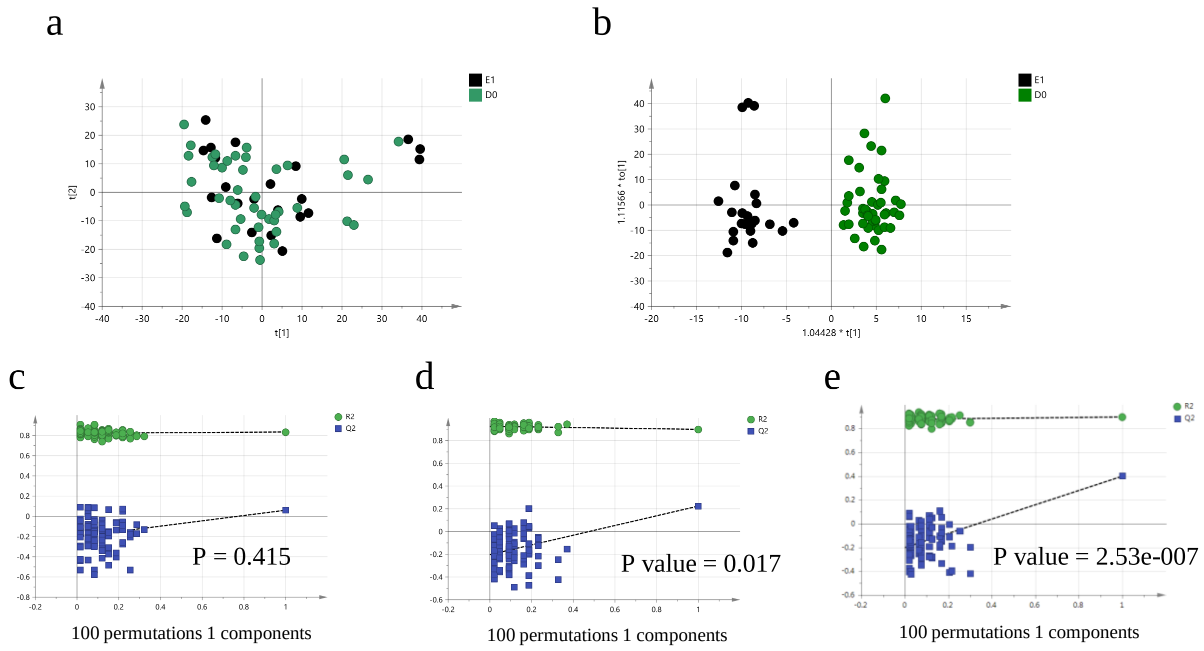

Supplementary figure 3. （a）PCA analysis of urine metabolome compared between baseline and first follow-up samples. (b) Score plot of OPLS-DA model based on metabolome between baseline and first follow-up samples. (c, d, e)100 permutation validation plot for the OPLS-DA model in first follow-up samples(c), second follow-up samples(d) and third follow-up samples(e).
