# Supplementary material for: Urinary metabolomic investigations in vitiligo patients
Source: Sci Rep. 2020 Oct 22;10:17989. doi: 10.1038/s41598-020-75135-0 (PMC7582886; doi:10.1038/s41598-020-75135-0)
Supplement: Supplementary file 3 — Supplementary Table 1. [file 41598_2020_75135_MOESM3_ESM.docx]

**Title: Urinary metabolomic investigations in vitiligo patients**

Author: Wei Liu^1^, Xiao-Yan Liu^2^, Yue-Tong Qian^1^, Dong-Dong Zhou^2^, Jia-Wei Liu^1^, Tian Chen^1^, Wei Sun^2*^, Dong-Lai Ma^1*^

1. Department of Dermatology, Peking Union Medical College Hospital, Chinese Academy of Medical Sciences, National Clinical Research Center for Skin and Immune Diseases, Beijing 100730, China.
2. Institute of Basic Medical Sciences, Chinese Academy of Medical Sciences, School of Basic Medicine, Peking Union Medical College, Beijing, 100005, China.

***** Correspondence: [mdonglai@sohu.com, 86-10-69151543 (DLM)](mailto:mdonglai@sohu.com,%2086-10-69151543%20(DLM));

[sunwei1018@sina.com](mailto:sunwei1018@sina.com), 86-10-69156995 (SW)

| **Vitiligo patient information** | | | | | | |
| --- | --- | --- | --- | --- | --- | --- |
| Patient NO. | Sex（Female/Male） | Age(years) | Disease phase | Disease type | Discovery cohort | Validation cohort |
| 1 | M | 15 | repigmentation | localized | √ |  |
| 2 | F | 19 | stable | sporadic | √ |  |
| 3 | F | 45 | active | sporadic | √ |  |
| 4 | M | 50 | stable | sporadic | √ |  |
| 5 | M | 13 | repigmentation | segmental | √ |  |
| 6 | M | 18 | repigmentation | sporadic | √ |  |
| 7 | M | 18 | active | sporadic | √ |  |
| 8 | F | 46 | stable | sporadic | √ |  |
| 9 | M | 64 | active | sporadic | √ |  |
| 10 | M | 34 | repigmentation | sporadic | √ |  |
| 11 | F | 6 | active | localized | √ |  |
| 12 | F | 7 | active | sporadic | √ |  |
| 13 | F | 24 | stable | localized | √ |  |
| 14 | F | 15 | repigmentation | sporadic | √ |  |
| 15 | F | 13 | active | sporadic | √ |  |
| 16 | M | 12 | active | sporadic | √ |  |
| 17 | M | 24 | active | sporadic | √ |  |
| 18 | F | 27 | stable | segmental | √ |  |
| 19 | F | 9 | stable | localized | √ |  |
| 20 | M | 43 | active | sporadic | √ |  |
| 21 | F | 53 | active | sporadic | √ |  |
| 22 | M | 23 | active | sporadic | √ |  |
| 23 | M | 38 | active | generalized | √ |  |
| 24 | F | 10 | active | sporadic | √ |  |
| 25 | F | 36 | active | localized | √ |  |
| 26 | M | 66 | stable | sporadic | √ |  |
| 27 | F | 43 | repigmentation | sporadic | √ |  |
| 28 | F | 40 | stable | localized | √ |  |
| 29 | M | 48 | active | sporadic | √ |  |
| 30 | F | 52 | repigmentation | sporadic | √ |  |
| 31 | F | 25 | stable | sporadic | √ |  |
| 32 | F | 50 | repigmentation | acrofacial | √ |  |
| 33 | M | 47 | repigmentation | acrofacial | √ |  |
| 34 | F | 45 | active | localized | √ |  |
| 35 | M | 11 | repigmentation | sporadic | √ |  |
| 36 | M | 27 | stable | localized | √ |  |
| 37 | M | 18 | repigmentation | localized | √ |  |
| 38 | M | 33 | active | sporadic | √ |  |
| 39 | F | 37 | repigmentation | sporadic | √ |  |
| 40 | M | 23 | repigmentation | sporadic | √ |  |
| 41 | M | 48 | repigmentation | sporadic | √ |  |
| 42 | F | 9 | repigmentation | segmental | √ |  |
| 43 | M | 9 | stable | localized | √ |  |
| 44 | F | 29 | active | localized | √ |  |
| 45 | M | 21 | stable | localized | √ |  |
| 46 | M | 12 | repigmentation | sporadic | √ |  |
| 47 | M | 8 | stable | sporadic | √ |  |
| 48 | F | 24 | repigmentation | sporadic | √ |  |
| 49 | M | 10 | stable | sporadic | √ |  |
| 50 | F | 15 | stable | sporadic | √ |  |
| 51 | M | 35 | stable | localized | √ |  |
| 52 | M | 14 | active | sporadic | √ |  |
| 53 | F | 24 | repigmentation | sporadic | √ |  |
| 54 | M | 5 | active | sporadic | √ |  |
| 55 | F | 5 | active | localized | √ |  |
| 56 | F | 30 | active | sporadic | √ |  |
| 57 | M | 11 | active | localized | √ |  |
| 58 | M | 24 | stable | sporadic | √ |  |
| 59 | F | 28 | stable | localized | √ |  |
| 60 | M | 18 | repigmentation | sporadic | √ |  |
| 61 | F | 12 | repigmentation | sporadic | √ |  |
| 62 | F | 43 | stable | localized | √ |  |
| 63 | M | 9 | stable | localized | √ |  |
| 64 | M | 27 | active | segmental | √ |  |
| 65 | M | 29 | active | sporadic | √ |  |
| 66 | M | 10 | active | sporadic | √ |  |
| 67 | F | 38 | stable | sporadic | √ |  |
| 68 | M | 10 | repigmentation | genernalized | √ |  |
| 69 | M | 14 | active | sporadic | √ |  |
| 70 | M | 6 | stable | localized | √ |  |
| 71 | M | 8 | repigmentation | sporadic | √ |  |
| 72 | M | 24 | repigmentation | localized | √ |  |
| 73 | M | 7 | repigmentation | localized | √ |  |
| 74 | F | 35 | stable | sporadic | √ |  |
| 75 | M | 19 | repigmentation | sporadic | √ |  |
| 76 | F | 34 | stable | localized | √ |  |
| 77 | F | 6 | repigmentation | segmental | √ |  |
| 78 | M | 11 | active | sporadic | √ |  |
| 79 | M | 46 | stable | sporadic | √ |  |
| 80 | F | 30 | repigmentation | sporadic | √ |  |
| 81 | M | 30 | repigmentation | acrofacial | √ |  |
| 82 | F | 36 | stable | sporadic | √ |  |
| 83 | F | 27 | repigmentation | localized | √ |  |
| 84 | F | 26 | active | sporadic | √ |  |
| 85 | F | 42 | active | sporadic | √ |  |
| 86 | M | 20 | active | segmental | √ |  |
| 87 | M | 5 | active | sporadic | √ |  |
| 88 | F | 46 | active | sporadic | √ |  |
| 89 | F | 17 | active | sporadic | √ |  |
| 90 | M | 4 | active | sporadic | √ |  |
| 91 | M | 37 | active | sporadic | √ |  |
| 92 | M | 25 | stable | localized | √ |  |
| 93 | F | 17 | active | segmental | √ |  |
| 94 | F | 11 | stable | sporadic | √ |  |
| 95 | M | 13 | stable | sporadic | √ |  |
| 96 | M | 28 | active | sporadic | √ |  |
| 97 | F | 9 | repigmentation | localized | √ |  |
| 98 | F | 10 | stable | localized | √ |  |
| 99 | M | 22 | stable | acrofacial | √ |  |
| 100 | F | 26 | stable | generalized | √ |  |
| 101 | F | 13 | stable | localized | √ |  |
| 102 | M | 19 | active | sporadic | √ |  |
| 103 | M | 17 | active | sporadic | √ |  |
| 104 | F | 12 | repigmentation | sporadic | √ |  |
| 105 | M | 17 | stable | sporadic | √ |  |
| 106 | F | 11 | repigmentation | sporadic | √ |  |
| 107 | F | 22 | stable | localized | √ |  |
| 108 | M | 16 | repigmentation | sporadic | √ |  |
| 109 | M | 24 | repigmentation | sporadic | √ |  |
| 110 | M | 15 | repigmentation | sporadic | √ |  |
| 111 | M | 44 | repigmentation | sporadic | √ |  |
| 112 | M | 12 | repigmentation | localized | √ |  |
| 113 | M | 11 | repigmentation | acrofacial | √ |  |
| 114 | F | 20 | repigmentation | localized | √ |  |
| 115 | M | 41 | active | localized | √ |  |
| 116 | M | 27 | repigmentation | sporadic | √ |  |
| 117 | M | 14 | active | sporadic | √ |  |
| 118 | F | 39 | active | sporadic | √ |  |
| 119 | M | 10 | active | generalized | √ |  |
| 120 | M | 20 | repigmentation | localized | √ |  |
| 121 | F | 14 | stable | sporadic | √ |  |
| 122 | F | 7 | stable | sporadic | √ |  |
| 123 | M | 10 | active | sporadic | √ |  |
| 124 | M | 9 | stable | localized | √ |  |
| 125 | M | 9 | active | sporadic | √ |  |
| 126 | M | 34 | active | sporadic | √ |  |
| 127 | F | 11 | active | sporadic | √ |  |
| 128 | F | 20 | stable | localized | √ |  |
| 129 | F | 11 | repigmentation | sporadic | √ |  |
| 130 | M | 21 | stable | sporadic | √ |  |
| 131 | F | 16 | active | sporadic | √ |  |
| 132 | M | 25 | repigmentation | acrofacial | √ |  |
| 133 | F | 17 | repigmentation | localized | √ |  |
| 134 | F | 38 | stable | sporadic | √ |  |
| 135 | M | 26 | active | sporadic | √ |  |
| 136 | F | 14 | repigmentation | segmental | √ |  |
| 137 | F | 16 | repigmentation | localized | √ |  |
| 138 | M | 10 | repigmentation | sporadic | √ |  |
| 139 | F | 30 | active | sporadic | √ |  |
| 140 | M | 27 | active | sporadic | √ |  |
| 141 | M | 16 | stable | sporadic | √ |  |
| 142 | M | 18 | active | sporadic | √ |  |
| 143 | M | 24 | stable | segmental | √ |  |
| 144 | M | 4 | active | sporadic | √ |  |
| 145 | F | 15 | repigmentation | sporadic | √ |  |
| 146 | M | 27 | active | sporadic | √ |  |
| 147 | M | 11 | repigmentation | sporadic | √ |  |
| 148 | M | 12 | repigmentation | sporadic | √ |  |
| 149 | F | 36 | repigmentation | sporadic | √ |  |
| 150 | F | 51 | repigmentation | sporadic | √ |  |
| 151 | F | 33 | repigmentation | localized | √ |  |
| 152 | M | 10 | active | localized | √ |  |
| 153 | F | 60 | active | sporadic | √ |  |
| 154 | F | 45 | active | sporadic | √ |  |
| 155 | F | 30 | active | sporadic | √ |  |
| 156 | F | 17 | repigmentation | sporadic | √ |  |
| 157 | F | 13 | active | sporadic | √ |  |
| 158 | F | 10 | repigmentation | sporadic | √ |  |
| 159 | M | 15 | repigmentation | sporadic | √ |  |
| 160 | M | 18 | repigmentation | sporadic | √ |  |
| 161 | M | 11 | repigmentation | localized | √ |  |
| 162 | F | 13 | repigmentation | segmental | √ |  |
| 163 | M | 19 | stable | sporadic | √ |  |
| 164 | M | 8 | stable | sporadic | √ |  |
| 165 | M | 20 | stable | sporadic | √ |  |
| 166 | M | 17 | active | Generalized | √ |  |
| 167 | M | 16 | stable | sporadic | √ |  |
| 168 | M | 8 | active | localized | √ |  |
| 169 | M | 18 | repigmentation | sporadic | √ |  |
| 170 | F | 48 | repigmentation | sporadic | √ |  |
| 171 | F | 29 | stable | sporadic | √ |  |
| 172 | M | 17 | repigmentation | localized | √ |  |
| 173 | F | 12 | repigmentation | sporadic | √ |  |
| 174 | M | 15 | active | segmental | √ |  |
| 175 | F | 15 | stable | localized | √ |  |
| 176 | M | 14 | active | sporadic | √ |  |
| 177 | M | 20 | repigmentation | localized | √ |  |
| 178 | F | 60 | active | sporadic | √ |  |
| 179 | M | 19 | repigmentation | localized | √ |  |
| 180 | M | 12 | repigmentation | localized | √ |  |
| 181 | F | 47 | stable | localized | √ |  |
| 182 | F | 42 | stable | acrofacial | √ |  |
| 183 | M | 10 | active | sporadic | √ |  |
| 184 | M | 7 | active | sporadic | √ |  |
| 185 | F | 56 | stable | localized | √ |  |
| 186 | F | 28 | active | sporadic | √ |  |
| 187 | M | 23 | stable | sporadic | √ |  |
| 188 | F | 19 | repigmentation | localized | √ |  |
| 189 | F | 24 | repigmentation | localized | √ |  |
| 190 | M | 18 | repigmentation | acrofacial | √ |  |
| 191 | M | 21 | stable | sporadic | √ |  |
| 192 | M | 30 | repigmentation | localized | √ |  |
| 193 | M | 16 | stable | localized | √ |  |
| 194 | F | 37 | active | localized | √ |  |
| 195 | F | 23 | active | sporadic | √ |  |
| 196 | F | 8 | repigmentation | localized | √ |  |
| 197 | F | 12 | active | acrofacial | √ |  |
| 198 | M | 17 | repigmentation | sporadic | √ |  |
| 199 | M | 30 | active | sporadic | √ |  |
| 200 | M | 19 | repigmentation | sporadic | √ |  |
| 201 | M | 8 | active | localized | √ |  |
| 202 | M | 12 | stable | sporadic | √ |  |
| 203 | M | 24 | stable | sporadic | √ |  |
| 204 | F | 27 | repigmentation | sporadic | √ |  |
| 205 | M | 17 | active | sporadic | √ |  |
| 206 | F | 46 | repigmentation | sporadic | √ |  |
| 207 | F | 28 | active | segmental | √ |  |
| 208 | F | 21 | stable | sporadic | √ |  |
| 209 | M | 19 | stable | sporadic | √ |  |
| 210 | F | 48 | repigmentation | sporadic | √ |  |
| 211 | M | 22 | stable | sporadic | √ |  |
| 212 | M | 27 | stable | generalized |  | √ |
| 213 | M | 12 | active | sporadic |  | √ |
| 214 | F | 51 | active | localized |  | √ |
| 215 | F | 18 | stable | sporadic |  | √ |
| 216 | F | 30 | active | sporadic |  | √ |
| 217 | F | 38 | active | localized |  | √ |
| 218 | F | 37 | active | generalized |  | √ |
| 219 | F | 12 | repigmentation | sporadic |  | √ |
| 220 | F | 17 | repigmentation | localized |  | √ |
| 221 | M | 8 | stable | localized |  | √ |
| 222 | F | 26 | active | sporadic |  | √ |
| 223 | F | 44 | active | sporadic |  | √ |
| 224 | M | 7 | stable | sporadic |  | √ |
| 225 | F | 53 | active | sporadic |  | √ |
| 226 | F | 41 | repigmentation | sporadic |  | √ |
| 227 | M | 11 | active | sporadic |  | √ |
| 228 | M | 18 | repigmentation | segmental |  | √ |
| 229 | F | 16 | repigmentation | segmental |  | √ |
| 230 | F | 39 | repigmentation | sporadic |  | √ |
| 231 | F | 6 | repigmentation | localized |  | √ |
| 232 | M | 34 | stable | sporadic |  | √ |
| 233 | M | 24 | active | sporadic |  | √ |
| 234 | M | 51 | stable | sporadic |  | √ |
| 235 | F | 58 | active | sporadic |  | √ |
| 236 | F | 24 | stable | localized |  | √ |
| 237 | F | 28 | repigmentation | sporadic |  | √ |
| 238 | F | 46 | repigmentation | sporadic |  | √ |
| 239 | F | 45 | repigmentation | sporadic |  | √ |
| 240 | F | 26 | stable | segmental |  | √ |
| 241 | M | 48 | repigmentation | localized |  | √ |
| 242 | F | 42 | repigmentation | sporadic |  | √ |
| 243 | M | 17 | repigmentation | sporadic |  | √ |
| 244 | M | 22 | repigmentation | sporadic |  | √ |
| 245 | M | 43 | repigmentation | sporadic |  | √ |
| 246 | F | 20 | stable | sporadic |  | √ |
| 247 | F | 7 | repigmentation | localized |  | √ |
| 248 | F | 36 | stable | localized |  | √ |
| 249 | F | 4 | stable | segmental |  | √ |
| 250 | M | 36 | active | sporadic |  | √ |
| 251 | F | 10 | repigmentation | sporadic |  | √ |
| 252 | F | 35 | stable | sporadic |  | √ |
| 253 | F | 11 | stable | sporadic |  | √ |
| 254 | F | 63 | repigmentation | sporadic |  | √ |
| 255 | F | 24 | stable | generalized |  | √ |
| 256 | F | 46 | repigmentation | sporadic |  | √ |
| 257 | M | 30 | stable | segmental |  | √ |
| 258 | F | 30 | active | acrofacial |  | √ |
| 259 | F | 32 | active | localized |  | √ |
| 260 | M | 9 | active | sporadic |  | √ |
| 261 | M | 32 | active | sporadic |  | √ |
| 262 | F | 17 | stable | sporadic |  | √ |
| 263 | M | 47 | repigmentation | acrofacial |  | √ |
| 264 | F | 11 | stable | sporadic |  | √ |
| 265 | F | 15 | stable | segmental |  | √ |
| 266 | F | 9 | repigmentation | localized |  | √ |
| 267 | F | 13 | active | sporadic |  | √ |
| 268 | M | 11 | repigmentation | sporadic |  | √ |
| 269 | M | 25 | active | sporadic |  | √ |
| 270 | F | 20 | repigmentation | sporadic |  | √ |
| 271 | M | 8 | stable | localized |  | √ |
| 272 | F | 27 | active | sporadic |  | √ |
| 273 | M | 15 | repigmentation | sporadic |  | √ |
| 274 | M | 10 | repigmentation | sporadic |  | √ |
| 275 | M | 22 | active | sporadic |  | √ |
| 276 | F | 28 | repigmentation | sporadic |  | √ |
| 277 | M | 16 | stable | segmental |  | √ |
| 278 | M | 11 | repigmentation | localized |  | √ |
| 279 | F | 12 | stable | localized |  | √ |
| 280 | M | 8 | active | sporadic |  | √ |
| 281 | F | 14 | stable | localized |  | √ |
| 282 | M | 10 | active | generalized |  | √ |
| 283 | M | 15 | repigmentation | sporadic |  | √ |
| 284 | F | 23 | stable | localized |  | √ |
| 285 | F | 13 | stable | localized |  | √ |
| 286 | M | 40 | active | sporadic |  | √ |
| 287 | M | 18 | stable | sporadic |  | √ |
| 288 | M | 14 | repigmentation | sporadic |  | √ |
| 289 | F | 48 | active | sporadic |  | √ |
| 290 | M | 13 | active | localized |  | √ |
| 291 | M | 9 | stable | sporadic |  | √ |
| 292 | F | 12 | stable | sporadic |  | √ |
| 293 | M | 23 | repigmentation | sporadic |  | √ |
| 294 | M | 12 | stable | localized |  | √ |
| 295 | F | 8 | active | sporadic |  | √ |

| **Healthy control patients information** | | | | |
| --- | --- | --- | --- | --- |
| patient NO. | Sex（Female/Male） | Age(years) | Discovery cohort | Validation cohort |
| 1 | M | 4 | √ |  |
| 2 | M | 5 | √ |  |
| 3 | M | 7 | √ |  |
| 4 | M | 7 | √ |  |
| 5 | M | 7 | √ |  |
| 6 | M | 8 | √ |  |
| 7 | M | 8 | √ |  |
| 8 | M | 8 | √ |  |
| 9 | M | 8 | √ |  |
| 10 | M | 9 | √ |  |
| 11 | M | 9 | √ |  |
| 12 | M | 9 | √ |  |
| 13 | M | 10 | √ |  |
| 14 | M | 10 | √ |  |
| 15 | M | 10 | √ |  |
| 16 | M | 10 | √ |  |
| 17 | M | 10 | √ |  |
| 18 | M | 10 | √ |  |
| 19 | M | 11 | √ |  |
| 20 | M | 11 | √ |  |
| 21 | M | 11 | √ |  |
| 22 | M | 11 | √ |  |
| 23 | M | 12 | √ |  |
| 24 | M | 12 | √ |  |
| 25 | M | 12 | √ |  |
| 26 | M | 12 | √ |  |
| 27 | M | 13 | √ |  |
| 28 | M | 14 | √ |  |
| 29 | M | 14 | √ |  |
| 30 | M | 16 | √ |  |
| 31 | M | 16 | √ |  |
| 32 | M | 17 | √ |  |
| 33 | M | 17 | √ |  |
| 34 | M | 18 | √ |  |
| 35 | M | 18 | √ |  |
| 36 | M | 18 | √ |  |
| 37 | M | 18 | √ |  |
| 38 | M | 18 | √ |  |
| 39 | M | 21 | √ |  |
| 40 | M | 24 | √ |  |
| 41 | M | 24 | √ |  |
| 42 | M | 26 | √ |  |
| 43 | M | 26 | √ |  |
| 44 | M | 27 | √ |  |
| 45 | M | 30 | √ |  |
| 46 | M | 31 | √ |  |
| 47 | M | 31 | √ |  |
| 48 | M | 32 | √ |  |
| 49 | M | 36 | √ |  |
| 50 | M | 43 | √ |  |
| 51 | M | 46 | √ |  |
| 52 | M | 47 | √ |  |
| 53 | M | 47 | √ |  |
| 54 | M | 48 | √ |  |
| 55 | M | 50 | √ |  |
| 56 | M | 51 | √ |  |
| 57 | M | 64 | √ |  |
| 58 | M | 66 | √ |  |
| 59 | F | 6 | √ |  |
| 60 | F | 6 | √ |  |
| 61 | F | 9 | √ |  |
| 62 | F | 9 | √ |  |
| 63 | F | 11 | √ |  |
| 64 | F | 11 | √ |  |
| 65 | F | 12 | √ |  |
| 66 | F | 12 | √ |  |
| 67 | F | 12 | √ |  |
| 68 | F | 12 | √ |  |
| 69 | F | 13 | √ |  |
| 70 | F | 13 | √ |  |
| 71 | F | 14 | √ |  |
| 72 | F | 15 | √ |  |
| 73 | F | 15 | √ |  |
| 74 | F | 16 | √ |  |
| 75 | F | 17 | √ |  |
| 76 | F | 17 | √ |  |
| 77 | F | 18 | √ |  |
| 78 | F | 19 | √ |  |
| 79 | F | 21 | √ |  |
| 80 | F | 22 | √ |  |
| 81 | F | 24 | √ |  |
| 82 | F | 25 | √ |  |
| 83 | F | 25 | √ |  |
| 84 | F | 26 | √ |  |
| 85 | F | 27 | √ |  |
| 86 | F | 28 | √ |  |
| 87 | F | 28 | √ |  |
| 88 | F | 28 | √ |  |
| 89 | F | 30 | √ |  |
| 90 | F | 30 | √ |  |
| 91 | F | 32 | √ |  |
| 92 | F | 35 | √ |  |
| 93 | F | 36 | √ |  |
| 94 | F | 36 | √ |  |
| 95 | F | 37 | √ |  |
| 96 | F | 38 | √ |  |
| 97 | F | 39 | √ |  |
| 98 | F | 40 | √ |  |
| 99 | F | 42 | √ |  |
| 100 | F | 42 | √ |  |
| 101 | F | 43 | √ |  |
| 102 | F | 45 | √ |  |
| 103 | F | 46 | √ |  |
| 104 | F | 46 | √ |  |
| 105 | F | 47 | √ |  |
| 106 | F | 48 | √ |  |
| 107 | F | 48 | √ |  |
| 108 | F | 51 | √ |  |
| 109 | F | 52 | √ |  |
| 110 | F | 53 | √ |  |
| 111 | F | 58 | √ |  |
| 112 | F | 61 | √ |  |
| 113 | F | 63 | √ |  |
| 1 | M | 8 |  | √ |
| 2 | M | 8 |  | √ |
| 3 | M | 8 |  | √ |
| 4 | M | 9 |  | √ |
| 5 | M | 9 |  | √ |
| 6 | M | 9 |  | √ |
| 7 | M | 10 |  | √ |
| 8 | M | 10 |  | √ |
| 9 | M | 11 |  | √ |
| 10 | M | 11 |  | √ |
| 11 | M | 11 |  | √ |
| 12 | M | 12 |  | √ |
| 13 | M | 12 |  | √ |
| 14 | M | 13 |  | √ |
| 15 | M | 14 |  | √ |
| 16 | M | 15 |  | √ |
| 17 | M | 16 |  | √ |
| 18 | M | 17 |  | √ |
| 19 | M | 18 |  | √ |
| 20 | M | 18 |  | √ |
| 21 | M | 21 |  | √ |
| 22 | M | 25 |  | √ |
| 23 | M | 27 |  | √ |
| 24 | M | 30 |  | √ |
| 25 | M | 31 |  | √ |
| 26 | M | 32 |  | √ |
| 27 | M | 35 |  | √ |
| 28 | M | 44 |  | √ |
| 29 | M | 47 |  | √ |
| 30 | M | 48 |  | √ |
| 31 | M | 54 |  | √ |
| 32 | F | 6 |  | √ |
| 33 | F | 8 |  | √ |
| 34 | F | 9 |  | √ |
| 35 | F | 9 |  | √ |
| 36 | F | 10 |  | √ |
| 37 | F | 11 |  | √ |
| 38 | F | 11 |  | √ |
| 39 | F | 11 |  | √ |
| 40 | F | 12 |  | √ |
| 41 | F | 12 |  | √ |
| 42 | F | 13 |  | √ |
| 43 | F | 13 |  | √ |
| 44 | F | 14 |  | √ |
| 45 | F | 15 |  | √ |
| 46 | F | 15 |  | √ |
| 47 | F | 17 |  | √ |
| 48 | F | 17 |  | √ |
| 49 | F | 19 |  | √ |
| 50 | F | 20 |  | √ |
| 51 | F | 21 |  | √ |
| 52 | F | 21 |  | √ |
| 53 | F | 22 |  | √ |
| 54 | F | 25 |  | √ |
| 55 | F | 26 |  | √ |
| 56 | F | 27 |  | √ |
| 57 | F | 28 |  | √ |
| 58 | F | 28 |  | √ |
| 59 | F | 29 |  | √ |
| 60 | F | 29 |  | √ |
| 61 | F | 30 |  | √ |
| 62 | F | 30 |  | √ |
| 63 | F | 30 |  | √ |
| 64 | F | 33 |  | √ |
| 65 | F | 36 |  | √ |
| 66 | F | 37 |  | √ |
| 67 | F | 38 |  | √ |
| 68 | F | 40 |  | √ |
| 69 | F | 41 |  | √ |
| 70 | F | 42 |  | √ |
| 71 | F | 43 |  | √ |
| 72 | F | 44 |  | √ |
| 73 | F | 45 |  | √ |
| 74 | F | 47 |  | √ |
| 75 | F | 47 |  | √ |
| 76 | F | 48 |  | √ |
| 77 | F | 51 |  | √ |
| 78 | F | 53 |  | √ |
| 79 | F | 60 |  | √ |

**Demographics of active vitiligo patients in longitudinal cohort enrolled in this study**

|  | Self-control cohort (n=46) |
| --- | --- |
| Average Age (years) | 32.7±12.45 |
| Sex |  |
| Female | 19 |
| Male | 27 |
| Disease type |  |
| Localized | 4 |
| Sporadic | 33 |
| Generalized | 9 |
| Comorbidity |  |
| Hepatitis B | 1 |
| Hypertension | 2 |
| Hyperthyroidism | 1 |
| Hashimoto thyroiditis | 1 |
| Chronic gastritis | 1 |
| Patients with effective response  repigmentation |  |
| 1st follow-up visit | n=32 |
| 2nd follow-up visit | n=34 |
| 3rd follow-up visit | n=38 |
