# Supplementary material for: Urinary metabolomic investigations in vitiligo patients
Source: Sci Rep. 2020 Oct 22;10:17989. doi: 10.1038/s41598-020-75135-0 (PMC7582886; doi:10.1038/s41598-020-75135-0)
Supplement: Supplementary file 4 — Supplementary Table 2. [file 41598_2020_75135_MOESM4_ESM.docx]

**Title: Urinary metabolomic investigations in vitiligo patients**

Author: Wei Liu^1^, Xiao-Yan Liu^2^, Yue-Tong Qian^1^, Dong-Dong Zhou^2^, Jia-Wei Liu^1^, Tian Chen^1^, Wei Sun^2*^, Dong-Lai Ma^1*^

1. Department of Dermatology, Peking Union Medical College Hospital, Chinese Academy of Medical Sciences, National Clinical Research Center for Skin and Immune Diseases, Beijing 100730, China.
2. Institute of Basic Medical Sciences, Chinese Academy of Medical Sciences, School of Basic Medicine, Peking Union Medical College, Beijing, 100005, China.

***** Correspondence: [mdonglai@sohu.com, 86-10-69151543 (DLM)](mailto:mdonglai@sohu.com,%2086-10-69151543%20(DLM));

[sunwei1018@sina.com](mailto:sunwei1018@sina.com), 86-10-69156995 (SW)

Differentially expressed metabolites detected between vitiligo patients and healthy controls.

| Name | AUC | P value* | FC^#^ |
| --- | --- | --- | --- |
| Procaterol | 0.84283 | 8.92E-14 | -6.7918 |
| 11,13-Dihydrotaraxinic acid glucosyl ester | 0.82692 | 8.21E-11 | -11.66 |
| Benzoyl ecgonine | 0.82327 | 1.18E-08 | -4.06 |
| 5-2,3-Dihydroxy-3-methylbutyl-4-3,4-epoxy-4-methylpentanoyl-3,4-dihydroxy-2-isopentanoyl-2-cyclopenten-1-one | 0.8177 | 4.89E-17 | -1.5181 |
| Citalopram | 0.77687 | 1.24E-12 | -4.6511 |
| 1-3-Methylbutanoyl-6-apiosylglucose | 0.76832 | 1.58E-07 | -4.8974 |
| Rishitin | 0.76678 | 1.38E-10 | -1.3279 |
| 3,4,5-trihydroxy-6-6Z-7-hydroxy-6-phenylmethylideneheptyloxyoxane-2-carboxylic acid | 0.75701 | 5.89E-04 | -2.4042 |
| Desglymidodrine | 0.75238 | 1.24E-10 | -1.7241 |
| 6-2-2H-1,3-benzodioxol-5-ylethyl-4-hydroxy-5,6-dihydro-2H-pyran-2-one | 0.74779 | 5.37E-05 | -2.6713 |
| Batatasin III | 0.74749 | 0.001415 | -3.4334 |
| 5-3',4'-dihydroxyphenyl-gamma-valerolactone-3'-O-glucuronide | 0.73654 | 3.13E-13 | 1.0878 |
| 3-Methylazelaic acid | 0.73533 | 1.16E-07 | -3.3419 |
| 7alpha-hydroxy-3-oxochol-4-en-24-oic Acid | 0.72486 | 1.90E-07 | -1.3366 |
| Threoninyl-Proline | 0.72343 | 2.17E-07 | -1.618 |
| Capsianoside V | 0.72076 | 2.23E-08 | -1.6084 |
| Deoxyuridine | 0.71902 | 2.80E-06 | 1.1866 |
| 3,4-octadienoylglycine | 0.71737 | 4.83E-06 | -1.2497 |
| 3,4,5-trihydroxy-6-3-4-methoxyphenylpropoxyoxane-2-carboxylic acid | 0.71355 | 2.77E-05 | -1.3702 |
| 2,4,6,8-Decatetraenoic acid dehydropiperidide | 0.71334 | 0.040617 | -1.841 |
| Coproporphyrin IV | 0.71268 | 2.53E-04 | -3.0924 |
| 1,2,10-Trihydroxydihydro-trans-linalyl oxide 7-O-beta-D-glucopyranoside | 0.70919 | 0.003866 | -2.5054 |
| 1R,2R,3S,1'R-Nepetalinic acid | 0.70881 | 1.24E-08 | -1.0335 |
| Gravolenic acid | 0.7084 | 4.10E-05 | -2.0914 |
| Flunisolide | 0.70469 | 1.04E-06 | -1.4501 |
| 9-Hydroxy-10-O-D-glucuronoside-12Z-octadecenoate | 0.70017 | 1.09E-07 | -0.9532 |
| N-1-Deoxy-1-fructosylvaline | 0.69657 | 7.98E-09 | -0.83826 |
| N1,N10-Dicoumaroylspermidine | 0.69584 | 6.37E-06 | -1.2234 |
| alpha-Ionol O-arabinosyl-1-6-glucoside | 0.69385 | 4.02E-06 | -1.3521 |
| Z-13-Hexadecenoic acid | 0.69068 | 7.87E-07 | -1.2022 |
| Flurandrenolide | 0.69036 | 9.81E-06 | -1.7116 |
| L-Kynurenine | 0.68798 | 0.005832 | -1.2864 |
| 3-hydroxy-3-4-hydroxy-3-methoxyphenylpropanoic acid | 0.6864 | 1.06E-04 | -3.0832 |
| 6-Acetyl-2,3-dihydro-2-hydroxymethyl-41H-pyridinone | 0.68474 | 0.20782 | -0.87922 |
| Anomurine | 0.67861 | 0.034581 | -0.86602 |
| 4-hydroxy-3-3-methylbut-2-en-1-ylbenzoic acid | 0.67826 | 0.2652 | 2.6813 |
| MG00/1846Z,9Z,12Z,15Z/00 | 0.67717 | 6.85E-05 | -1.1257 |
| 2-Furoylglycine | 0.67354 | 4.60E-05 | -1.0797 |
| gamma-Glutamyl-S-1-propenylcysteine sulfoxide | 0.67273 | 0.002829 | 0.80618 |
| L-Agaridoxin | 0.67152 | 2.67E-05 | -1.2351 |
| 5-Hydroxy-p-mentha-6,8-dien-2-one | 0.67078 | 3.89E-04 | -1.9855 |
| 2-octenoylglycine | 0.66735 | 0.082006 | -3.9623 |
| Tryptophol xylosyl-1-6-glucoside | 0.66656 | 2.98E-04 | -1.4873 |
| 3E,5Z-1,3,5-Tridecatriene-7,9,11-triyne | 0.66531 | 0.15584 | -0.74752 |
| Oleoside dimethyl ester | 0.66406 | 1.58E-07 | -2.1084 |
| Austalide L | 0.65667 | 0.002919 | -0.75 |
| cis-4-Decenedioic acid | 0.653 | 6.51E-08 | -1.6501 |
| Tanacetin | 0.65249 | 7.85E-07 | 0.76464 |
| Agamanone | 0.65072 | 6.70E-05 | -2.0881 |
| E-2-Methyl-2-buten-1-ol O-beta-D-Glucopyranoside | 0.64745 | 0.001726 | -0.94276 |
| Dextrorphan O-glucuronide | 0.64275 | 0.29493 | -2.2865 |
| Linalyl anthranilate | 0.64168 | 0.108 | -0.78246 |
| Isocarboxazid | 0.63658 | 0.017653 | -0.70517 |
| Cefadroxil | 0.63224 | 0.59306 | -1.9547 |
| 9-Methyluric acid | 0.62809 | 1.28E-06 | 0.71623 |
| Deoxyloganic acid | 0.62783 | 1.73E-06 | -0.93069 |
| Tipredane | 0.62526 | 1.73E-04 | -1.1553 |
| Avenanthramide A2 | 0.62489 | 8.25E-05 | -1.8462 |
| Talaromycin A | 0.62453 | 0.01616 | -0.7717 |
| Ganoderic acid epsilon | 0.62394 | 0.064027 | 2.5254 |
| Travoprost | 0.61949 | 0.001562 | -0.91308 |
| 2-4-Hydroxy-3,5-dimethoxyphenylethanol 4'-glucoside | 0.60726 | 0.011957 | -0.86672 |
| 2-Aminonicotinic acid | 0.60507 | 0.18604 | -3.1092 |
| D-Urobilinogen | 0.60048 | 0.011269 | -0.97523 |
| S-Adenosylmethioninamine | 0.59855 | 0.20691 | -1.4582 |
| Salicylic acid beta-D-glucoside | 0.59695 | 0.47535 | -2.5785 |
| 2,3-Butanediol glucoside | 0.59328 | 0.18236 | -1.5506 |
| 6-5-1-carboxyethyl-4-hydroxy-2-methoxyphenoxy-3,4,5-trihydroxyoxane-2-carboxylic acid | 0.59076 | 6.72E-05 | -1.4019 |
| Dehydroepiandrosterone sulfate | 0.58933 | 0.082145 | -1.738 |
| S-Argpyrimidine | 0.56336 | 0.35358 | -2.2058 |
| 1x,2x-Guaiacylglycerol 2-glucoside | 0.53386 | 2.22E-04 | -0.54958 |

*P value: Benjamini-Hochberg correction was applied throughout to account for multiple test comparisons. Cutoff of FDR 0.05 was applied.

# Fold change.
