# Supplementary material for: Urinary metabolomic investigations in vitiligo patients
Source: Sci Rep. 2020 Oct 22;10:17989. doi: 10.1038/s41598-020-75135-0 (PMC7582886; doi:10.1038/s41598-020-75135-0)
Supplement: Supplementary file 5 — Supplementary Table 3. [file 41598_2020_75135_MOESM5_ESM.docx]

**Title: Urinary metabolomic investigations in vitiligo patients**

Author: Wei Liu^1^, Xiao-Yan Liu^2^, Yue-Tong Qian^1^, Dong-Dong Zhou^2^, Jia-Wei Liu^1^, Tian Chen^1^, Wei Sun^2*^, Dong-Lai Ma^1*^

1. Department of Dermatology, Peking Union Medical College Hospital, Chinese Academy of Medical Sciences, National Clinical Research Center for Skin and Immune Diseases, Beijing 100730, China.
2. Institute of Basic Medical Sciences, Chinese Academy of Medical Sciences, School of Basic Medicine, Peking Union Medical College, Beijing, 100005, China.

***** Correspondence: [mdonglai@sohu.com, 86-10-69151543 (DLM)](mailto:mdonglai@sohu.com,%2086-10-69151543%20(DLM));

[sunwei1018@sina.com](mailto:sunwei1018@sina.com), 86-10-69156995 (SW)

**41 differential metabolites detected between baseline and second follow-up samples**

| Name | AUC | P value* | Fold change |
| --- | --- | --- | --- |
| (1R,2R,3S,1'R)-Nepetalinic acid | 0.77295 | 7.77E-04 | 4.116 |
| (1S,2S,4R,8R)-p-Menthane-1,2,8,9-tetrol | 0.71437 | 2.13E-02 | 3.459 |
| (1x,2x)-Guaiacylglycerol 3-glucoside | 0.75121 | 2.92E-03 | 4.542 |
| (S)-3-Hydroxy-N-methylcoclaurine | 0.74849 | 4.67E-03 | 2.874 |
| (卤)-1-(4-Methylphenyl)ethanol | 0.77657 | 6.56E-04 | 7.254 |
| 1-(2,4-Dihydroxyphenyl)-1-butanone | 0.79952 | 1.48E-04 | 3.008 |
| 1,1'-Ethylidenebistryptophan | 0.7663 | 1.13E-03 | 2.901 |
| 1,4-octadienoylglycine | 0.78684 | 3.45E-04 | 4.846 |
| 16a-Hydroxydehydroisoandrosterone | 0.7663 | 1.80E-03 | 4.001 |
| 17-phenyl-18,19,20-trinor-prostaglandin D2 | 0.75362 | 2.57E-03 | 3.653 |
| 2-(4-Hydroxy-3,5-dimethoxyphenyl)ethanol 4'-glucoside | 0.77657 | 6.56E-04 | 11.028 |
| 2,3-Dimethyl-2-cyclohexen-1-one | 0.83998 | 2.26E-05 | 10.365 |
| 2-[2-hydroxy-3-(3-hydroxy-4-methoxyphenyl)propyl]benzene-1,3,5-triol | 0.70048 | 4.39E-02 | 0.4 |
| 3-(4-Hydroxy-3-methoxyphenyl)-1,2-propanediol | 0.77778 | 6.31E-04 | 4.917 |
| 3,4-Methylenedioxyamphetamine | 0.77717 | 6.43E-04 | 3.88 |
| 3-Amino-1,4-dimethyl-5H-pyrido[4,3-b]indole | 0.75211 | 3.87E-03 | 20.557 |
| 3-Hydroxy-6,8-dimethoxy-7(11)-eremophilen-12,8-olide | 0.81854 | 9.35E-05 | 3.308 |
| 3-Hydroxy-p-mentha-1,8-dien-7-al | 0.85628 | 2.40E-05 | 29.514 |
| 4-Isopropyl-3-cyclohexene-1-carboxylic acid | 0.78442 | 4.08E-04 | 10.106 |
| 4-Methoxy-17beta-estradiol | 0.77506 | 1.11E-03 | 2.289 |
| 5-[2-(2-hydroxyphenyl)ethyl]-2,3-dimethoxyphenol | 0.84209 | 3.17E-05 | 4.082 |
| 5-Hydroxysebacate | 0.80707 | 1.88E-04 | 9.5 |
| 6-Epi-7-isocucurbic acid glucoside | 0.76147 | 1.58E-03 | 3.175 |
| 6-Hydroxyfluvastatin | 0.82971 | 2.70E-05 | 3.362 |
| Acetylsalvipisone | 0.73732 | 6.29E-03 | 0.455 |
| alpha-Carboxy-delta-nonalactone | 0.82126 | 7.72E-05 | 5.626 |
| Avenanthramide 2 | 0.75302 | 2.64E-03 | 4.977 |
| Avenanthramide A2 | 0.83424 | 3.94E-05 | 10.226 |
| Benzyl O-[arabinofuranosyl-(1->6)-glucoside] | 0.76812 | 1.63E-03 | 6.064 |
| Dihydroconiferin | 0.81703 | 4.17E-05 | 16.185 |
| Gossyvertin | 0.85809 | 5.22E-06 | 8.37 |
| N1,N10-Dicoumaroylspermidine | 0.7026 | 4.05E-02 | 0.433 |
| N-Acetylaminooctanoic acid | 0.73249 | 1.03E-02 | 10.156 |
| Nordihydrocapsiate | 0.70471 | 3.28E-02 | 2.921 |
| Phenethyl rutinoside | 0.79589 | 1.83E-04 | 0.486 |
| Poly-N-acetyllactosamine | 0.78291 | 7.20E-04 | 2.959 |
| Procaterol | 0.84692 | 2.70E-05 | 3.725 |
| Salbutamol 4-O-sulfate | 0.78321 | 7.20E-04 | 7.33 |
| S-Furanopetasitin | 0.70562 | 3.50E-02 | 0.409 |
| Simmondsin | 0.82065 | 3.65E-05 | 4.88 |
| Takakin | 0.70562 | 3.50E-02 | 4.482 |

*P value: Benjamini-Hochberg correction was applied throughout to account for multiple test comparisons. Cutoff of FDR 0.05 was applied

**42 differential metabolites detected between baseline and third follow-up samples**

| Name | AUC | P value* | Fold change |
| --- | --- | --- | --- |
| (1R,2R,3S,1'R)-Nepetalinic acid | 0.73741 | 5.49E-03 | 5.622 |
| (1x,2x)-Guaiacylglycerol 3-glucoside | 0.75458 | 2.17E-03 | 3.831 |
| (4S,8R)-8,9-Dihydroxy-p-menth-1(6)-en-2-one | 0.83581 | 2.21E-05 | 32.9 |
| (S)-3-Hydroxy-N-methylcoclaurine | 0.72941 | 8.18E-03 | 2.449 |
| 1,4-octadienoylglycine | 0.73112 | 7.58E-03 | 3.722 |
| 2-[4,6-dihydroxy-2-methoxy-3-(3-methylbut-2-en-1-yl)phenyl]-1-(2,4,5-trihydroxyphenyl)ethan-1-one | 0.78719 | 2.76E-04 | 0.468 |
| 2-Methylbenzoic acid | 0.76945 | 8.29E-04 | 6.029 |
| 3,4,5-trihydroxy-6-(2-oxoethoxy)oxane-2-carboxylic acid | 0.73055 | 7.79E-03 | 2.503 |
| 3,4,5-trihydroxy-6-{[4-hydroxy-5-(3-methoxyphenyl)pentanoyl]oxy}oxane-2-carboxylic acid | 0.75229 | 2.43E-03 | 2.878 |
| 3,4,5-trihydroxy-6-{3-hydroxy-4-[(2E)-4-hydroxy-3-methylbut-2-en-1-yl]phenoxy}oxane-2-carboxylic acid | 0.70166 | 3.33E-02 | 2.436 |
| 3,4-Methylenedioxyamphetamine | 0.81465 | 5.67E-05 | 3.041 |
| 3-[3,4-dihydroxy-5-(3-methylbut-2-en-1-yl)phenyl]-1-(2,4-dihydroxyphenyl)propan-1-one | 0.75229 | 2.43E-03 | 3.347 |
| 3-Amino-1,4-dimethyl-5H-pyrido[4,3-b]indole | 0.76716 | 9.55E-04 | 10.491 |
| 3-carboxy-4-methyl-5-pentyl-2-furanpropanoic acid | 0.72941 | 8.18E-03 | 2.115 |
| 3-Hydroxy-6,8-dimethoxy-7(11)-eremophilen-12,8-olide | 0.79462 | 1.78E-04 | 3.047 |
| 3-Hydroxy-p-mentha-1,8-dien-7-al | 0.85011 | 2.21E-05 | 28.79 |
| 5-(2,3-Dihydroxy-3-methylbutyl)-4-(3,4-epoxy-4-methylpentanoyl)-3,4-dihydroxy-2-isopentanoyl-2-cyclopenten-1-one | 0.71968 | 1.62E-02 | 0.435 |
| 5-(3',5'-Dihydroxyphenyl)-gamma-valerolactone-O-glucuronide-O-methyl | 0.71224 | 1.95E-02 | 0.471 |
| 5-Hydroxysebacate | 0.78976 | 4.42E-04 | 8.012 |
| 6-[(E)-2-(4-hydroxyphenyl)ethenyl]-4-methoxy-5,6-dihydro-2H-pyran-2-one | 0.73112 | 9.32E-03 | 4.324 |
| 6-Hydroxy-2,6-dimethyl-2,7-octadien-4-one | 0.77117 | 7.43E-04 | 7.955 |
| 8-Hydroxycarteolol | 0.80292 | 2.15E-04 | 3.556 |
| Acetylsalvipisone | 0.74771 | 3.07E-03 | 0.394 |
| alpha-Carboxy-delta-nonalactone | 0.79863 | 1.38E-04 | 5.884 |
| Avenanthramide A2 | 0.76688 | 1.59E-03 | 7.951 |
| Dehydroepiandrosterone | 0.746 | 4.61E-03 | 4.845 |
| Deoxyloganic acid | 0.72254 | 1.20E-02 | 6.618 |
| Dihydroconiferin | 0.83238 | 2.21E-05 | 12.456 |
| Dihydroferuloylglycine | 0.70538 | 2.56E-02 | 2.809 |
| Diosmetin | 0.70166 | 3.33E-02 | 2.481 |
| Dopamine | 0.8135 | 6.03E-05 | 3.552 |
| Gossyvertin | 0.81693 | 9.80E-05 | 3.688 |
| Gravolenic acid | 0.8238 | 7.38E-05 | 6.582 |
| Histidinyl-Arginine | 0.70938 | 2.20E-02 | 0.37 |
| Isoaustin | 0.87128 | 2.13E-05 | 5.077 |
| Meclizine | 0.78604 | 2.95E-04 | 0.466 |
| N-Acetylaminooctanoic acid | 0.70395 | 3.06E-02 | 4.771 |
| N-Acetylhistidine | 0.75172 | 2.46E-03 | 0.32 |
| N-cis-Feruloyltyramine | 0.75114 | 2.53E-03 | 0.5 |
| Syndesine | 0.82723 | 2.49E-05 | 4.685 |
| Threoninyl-Proline | 0.73341 | 6.81E-03 | 3.038 |
| Topotecan | 0.81608 | 1.02E-04 | 2.931 |

*P value: Benjamini-Hochberg correction was applied throughout to account for multiple test comparisons. Cutoff of FDR 0.05 was applied
